# Dapagliflozin Improves Angiogenesis after Hindlimb Ischemia through the PI3K-Akt-eNOS Pathway

**DOI:** 10.3390/biom14050592

**Published:** 2024-05-16

**Authors:** Li Han, Guoxin Ye, Wenjing Su, Yuankang Zhu, Wenqi Wu, Liangshi Hao, Jing Gao, Zhen Li, Fang Liu, Junli Duan

**Affiliations:** Department of Geriatrics, Xinhua Hospital Affiliated to Shanghai Jiao Tong University School of Medicine, Kongjiang Road 1665, Shanghai 200092, China; hanli@xinhuamed.com.cn (L.H.); yeguoxin8574@xinhuamed.com.cn (G.Y.); suwenjing@xinhuamed.com.cn (W.S.); 15258683336@sjtu.edu.cn (Y.Z.); 2020221624@stu.cqmu.edu.cn (W.W.); haoliangshi@sjtu.edu.cn (L.H.); gaojing-jane@alumni.sjtu.edu.cn (J.G.); lizhen8367@xinhuamed.com.cn (Z.L.)

**Keywords:** dapagliflozin, angiogenesis, PI3K-Akt-eNOS

## Abstract

Recently, the vascular protective effect of anti-diabetic agents has been receiving much attention. Sodium glucose cotransporter 2 (SGLT2) inhibitors had demonstrated reductions in cardiovascular (CV) events. However, the therapeutic effect of dapagliflozin on angiogenesis in peripheral arterial disease was unclear. This study aimed to explore the effect and mechanism of dapagliflozin on angiogenesis after hindlimb ischemia. We first evaluated the effect of dapagliflozin on post-ischemic angiogenesis in the hindlimbs of rats. Laser doppler imaging was used to detect the hindlimb blood perfusion. In addition, we used immunohistochemistry to detect the density of new capillaries after ischemia. The relevant signaling pathways of dapagliflozin affecting post-ischemic angiogenesis were screened through phosphoproteomic detection, and then the mechanism of dapagliflozin affecting post-ischemic angiogenesis was verified at the level of human umbilical vein endothelial cells (HUVECs). After subjection to excision of the left femoral artery, all rats were randomly distributed into two groups: the dapagliflozin group (left femoral artery resection, receiving intragastric feeding with dapagliflozin (1 mg/kg/d), for 21 consecutive days) and the model group, that is, the positive control group (left femoral artery resection, receiving intragastric feeding with citric acid–sodium citrate buffer solution (1 mg/kg/d), for 21 consecutive days). In addition, the control group, that is the negative control group (without left femoral artery resection, receiving intragastric feeding with citric acid–sodium citrate buffer solution (1 mg/kg/d), for 21 consecutive days) was added. At day 21 post-surgery, the dapagliflozin-treatment group had the greatest blood perfusion, accompanied by elevated capillary density. The results showed that dapagliflozin could promote angiogenesis after hindlimb ischemia. Then, the ischemic hindlimb adductor-muscle tissue samples from three rats of model group and dapagliflozin group were taken for phosphoproteomic testing. The results showed that the PI3K-Akt-eNOS signaling pathway was closely related to the effect of dapagliflozin on post-ischemic angiogenesis. Our study intended to verify this mechanism from the perspective of endothelial cells. In vitro, dapagliflozin enhanced the tube formation, migration, and proliferation of HUVECs under ischemic and hypoxic conditions. Additionally, the dapagliflozin administration upregulated the expression of angiogenic factors phosphorylated Akt (p-Akt) and phosphorylated endothelial nitric oxide synthase (p-eNOS), as well as vascular endothelial growth factor A (VEGFA), both in vivo and in vitro. These benefits could be blocked by either phosphoinositide 3-kinase (PI3K) or eNOS inhibitor. dapagliflozin could promote angiogenesis after ischemia. This effect might be achieved by promoting the activation of the PI3K-Akt-eNOS signaling pathway. This study provided a new perspective, new ideas, and a theoretical basis for the treatment of peripheral arterial disease.

## 1. Introduction

Peripheral arterial disease (PAD) is a common and serious cardiovascular disease, which is easily complicated by limb ischemia and cardiovascular adverse events. PAD, caused by narrowing of the arteries in the limbs, is increasing in incidence and prevalence as our population is ageing and as diabetes is becoming more prevalent [1]. Treatments for PAD include medications that reduce the increased risk of cardiovascular events and help improve blood flow, as well as endovascular or surgical repair or the bypass of blocked arteries [2]. Due to various limitations, a large proportion of PAD patients are not effectively and adequately treated, and have to undergo a brutal amputation [3]. Therefore, the search for safe and effective methods to promote the angiogenesis of ischemic sites has become one of the research hotspots in recent years [4]. At present, although there have been great changes in drug therapy, surgical bypass surgery and catheter interventional therapy for PAD, all kinds of treatment methods have their own strict indications [3]. For patients with serious comorbidities (such as diabetes, severe kidney failure or heart failure), surgery or interventional catheter therapy is often not suitable, and the effect of conventional drug therapy is not satisfactory. So, are there any new oral drugs that have a good effect on peripheral artery disease? In recent years, sodium glucose transporter 2 inhibitors (SGLT2is) have been approved for clinical use as a new class of hypoglycemic drugs, among which dapagliflozin, one of the SGLT2is, is currently approved for the treatment of non-diabetic patients with heart failure [5]. Dapagliflozin can significantly reduce the total number of cardiovascular deaths in patients with diabetes and chronic kidney disease, and has protective effects on cardio-renal vessels [6]. Does dapagliflozin have a protective effect on peripheral arteries? We intend to study the mechanism of dapagliflozin on angiogenesis after hindlimb ischemia. Dapagliflozin is a SGLT2i, a new class of hypoglycemic drugs, which can inhibit the absorption of glucose and sodium by the kidney [7,8]. In recent years, there has been evidence that dapagliflozin can significantly reduce the total number of deaths from cardiovascular causes in patients with diabetes and chronic kidney disease [9]. In addition, dapagliflozin may be beneficial for patients with non-diabetic heart failure [9]. Dapagliflozin has a protective effect on cardio-renal vessels, but its underlying mechanism has not been fully understood, and in recent years, studies have found that different types of SGLT2i have different effects on different target organs [6,9]. The underlying mechanisms of the cardiovascular benefits of SGLT2is remain unknown, but they do not appear to be mediated by differential improvements in blood glucose control [10]. While lowering blood pressure, arterial stiffness, body weight, visceral obesity, and/or intrarenal hemodynamics have been suggested to contribute to the cardiovascular benefits of SGLT2is, the direct effects of these drugs on vascular endothelial cell function have not been considered. The aim of this study was to investigate the effect and mechanism of dapagliflozin on angiogenesis after hindlimb ischemia [11,12]. 

## 2. Materials and Methods

### 2.1. Animals

SD male rats, aged 8 weeks, were purchased from Shanghai SLAC Laboratory Animal Co. Ltd. (Shanghai, China), housed in a temperature- and humidity-controlled room with a 12:12 h light–dark cycle, and provided with ad libitum access to water and food. All animal studies and operative procedures were approved by the Shanghai Jiao Tong University Animal Care and Use Committee. All surgeries were performed under anesthesia, and all efforts were made to minimize suffering.

### 2.2. Rat Hindlimb Ischemia Model and Treatments

Briefly, all rats were anesthetized by intraperitoneal injection of sodium pentobarbital (60 mg/kg) [13]. Unilateral hindlimb ischemia was surgically induced as previously described [14]. Surgery was performed by excising the femoral artery of the left hindlimb, while the corresponding right hindlimb was left without surgery and used as a self-control. Rats were then grouped randomly. Then, all rats were divided into 3 groups: (1) the dapagliflozin group, with left femoral artery resection, receiving intragastric feeding with dapagliflozin (1 mg/kg/d), for 21 consecutive days (*n* = 10); (2) the model group, that is, the positive control group: with left femoral artery resection, receiving intragastric feeding with citric acid–sodium citrate buffer solution (1 mg/kg/d), for 21 consecutive days (*n* = 10); and (3) the control group, that is, the negative control group: without left femoral artery resection, receiving intragastric feeding with citric acid–sodium citrate buffer solution (1 mg/kg/d), for 21 consecutive days (*n* = 10). The rats in the dapagliflozin group received intragastric feeding with dapagliflozin (1 mg/kg/d), for 21 consecutive days. Rats in model group and control group were given the same amount (1 mg/kg/d) of citric acid–sodium citrate buffer solution at the same time, for 21 consecutive days. All the treatments started from 24 h after surgery, and the administration lasted to the end of the experiment. Blood perfusion in the hindlimb was visualized and analyzed by laser doppler imager (Moor Instruments Ltd., Axminster, Devon, UK) at indicated time points. Prior to visualization, rats were anesthetized and the fur of the hindlimb region was depilated. The blood perfusion ratio was acquired by calculating the ratio between the ischemic hindlimb (left hindlimb) and the corresponding control (right hindlimb) [15,16,17], at six predetermined time points (before and immediately after surgery and at postoperative days 3, 7, 14 and 21). Laser doppler blood flow imaging of rat hindlimbs can observe the effect of dapagliflozin in promoting angiogenesis after hindlimb ischemia. CD31 immunohistochemical staining can reflect the role of dapagliflozin in promoting angiogenesis after hindlimb ischemia. Animals were euthanized after finishing laser doppler scanning on postoperative day 21. Blood samples and hindlimb adductor muscle tissue samples were collected for subsequent experiments.

### 2.3. General Condition and Blood Analysis

The three groups of rats were weighed at the beginning of the experiment, which is the day when the hindlimb ischemia model was established (d0), and at the end of the experiment, which was the 21st day after hindlimb ischemia surgery (d21), respectively, and the peripheral blood of the rats was collected for blood glucose measurement, liver and kidney function, serum sodium, blood potassium, glycated serum protein and other indicators. An automatic biochemical analyzer (HITACHI 7600-020) was used to measure biochemical indicators, including blood glucose, liver and kidney function, serum sodium and potassium, and glycated serum protein.

### 2.4. Immunohistochemistry Analysis

Animals were euthanized after finishing laser doppler scanning on postoperative day 21. Before euthanasia, blood was taken from the abdominal aorta and centrifuged at 3000 r/min for 10 min with a centrifuge radius of 8 cm. Supernatant was taken and placed in the refrigerator at −80 °C for storage. The thigh adductor muscle was immersed in 4% formaldehyde and embedded in paraffin for immunohistochemistry analysis. Capillary density in each cross-section (5 μm) was labeled by immunohistochemical staining [18]. Briefly, antigen retrieval and blocking procedures were followed by sequential incubation with goat anti-CD31 antibody (BD, Franklin Lakes, NJ, USA) and secondary antibody (Invitrogen, Carlsbad, CA, USA). Images were collected and analyzed under a microscope, and the average number of microvessels in each field of view was counted as the MVD value (pieces/HP).

### 2.5. Western Blotting Analysis (Tissue Protein)

Western blotting detected the expression of PI3K/Akt/eNOS signaling pathway proteins in the ischemic hindlimb muscle tissue of rats in each group. After extracting the total protein from the tissue, the concentration and purity were detected, followed by electrophoresis, and transferred to the membrane. Skim milk powder was sealed at room temperature. For 2 h, the added primary antibodies (anti-eNOS, anti-p-eNOS (Ser1177), anti-Akt, anti-p-Akt (Ser473), anti-vascular endothelial growth factor A (VEGFA) (Beyotime, Haimen, China)) and the internal reference (Anti-GAPDH (Cell Signaling Technology, Danvers, MA, USA)), were diluted 1:1000, respectively. The next day, the secondary antibody was added and incubated at room temperature for 1 h, the membrane was washed, and chemiluminescence was performed. Based on the gray value ratio of each protein band, the relative protein expression was calculated. Original western blots are available in the Supplementary Materials.

### 2.6. D Label-Free Phosphorylated Protein Spectrum

The ischemic hindlimb adductor muscle tissue samples from three rats of the model group and dapagliflozin group were taken for phosphoproteomic testing. The main steps of this experiment included quantification and quality inspection of the protein extracted from the original sample, FASP enzymatic hydrolysis and modified peptide enrichment, final on-machine detection and bioinformatics analysis of mass spectrometry data. Specifically we carried out the following: (1) we used homogenization and SDT lysis methods to extract protein from the original sample. First, we took a rat hindlimb muscle tissue sample, then added an appropriate amount of SDT lysis solution and transferred it to a Lysing Matrix A tube, and then used an MP homogenizer for ultrasonic homogenization. Pulp crushing was carried out (24 × 2, 6.0 M/S, 30 s) twice. After sonication, we placed the sample in a boiling water, bathed it for 10 min, then centrifuged it at 14,000× *g* for 15 min, took the supernatant, and filtered it with a 0.22 µm filter membrane, and collected the filtrate. We used the BCA method for protein quantification and aliquots, and the remaining samples could be stored at −80 °C. (2). We took 20 µg of each sample, added 6X loading buffer, bathed it in boiling water for 5 min, performed 12% separation gel SDS-PAGE electrophoresis (constant voltage 250 V, 40 min), and stained it with Coomassie Brilliant Blue to check the protein abundance. (3) We took 100 µg of protein lysate from each sample, added DTT to a final concentration of 100 mM, put it in a boiling water bath for 5 min, and cooled to room temperature. We added 200 μL UA buffer, mixed well, transferred it to a 30 kD ultrafiltration centrifuge tube, centrifuged it at 12,500× *g* for 15 min, and discarded the filtrate (we repeated this step once). We added 100 μL IAA buffer (100 mM IAA in UA), shook it at 600 rpm for 1 min, reacted it at room temperature in the dark for 30 min, and centrifuged it at 12,500× *g* for 15 min. We added 100 μL UA buffer and centrifuged at 12,500× *g* for 15 min. We repeated this step twice. We added 100 μL of 50 mM NH_4_HCO_3_ solution, centrifuged it at 12,500× *g* for 15 min, and repeated this step twice. We replaced the collection tube with a new one, added 40 μL Trypsin buffer (4 μg Trypsin in 40 μL 50 mM NH_4_HCO_3_ solution), shook it at 600 rpm for 1 min, and placed it at 37 °C for 16–18 h. We centrifuged it at 12,500× *g* for 15 min; we added 40 μL of 50 mM NH_4_HCO_3_ solution, centrifuged it at 12,500× *g* for 15 min, and collected the filtrate. C18 Cartridge was used to desalt the peptides. After the peptides were lyophilized, 40 μL of 0.1% formic acid solution was added to reconstitute them. The peptides were quantified by a UV spectrophotometer (OD280). (4) The peptides were desalted using a C18 Cartridge (Waters, WAT023590) column, lyophilized and then enriched with phosphorylated peptides. (5) Each sample was separated using the Easy NLC system at nanoscale flow rates. Buffer solution A was a 0.1% formic acid aqueous solution, and buffer solution B was a 0.1% formic acid acetonitrile aqueous solution (acetonitrile is 80%). The column was equilibrated with 100% A solution, and the sample was sent to the analytical column (Termo Fisher Scientific, Acclaim Pepmap RSLC 50 μm × 15 cm, Nano Viper, P/N164943) using an autosampler at a flow rate of 300 nL/min. After chromatographic separation, the samples were analyzed by mass spectrometry using the PASEF mode of a timsTOF Pro (Bruker, Bremen, Germany) mass spectrometer. The analysis time was 90 min, the detection method was positive ion, the precursor ion scanning range was 100–1700 m/z, the range of ion mobility 1/K0 was 0.75–1.4 V·s/cm^2^, the ion accumulation or release time was 100 ms, the ion utilization rate was 100%, the capillary voltage was 1500 V, the drying gas speed was 3 L/min, and the drying temperature was 180 °C. The settings of PASEF were the following: 10 MS/MS scans (total cycle time was 1.16 s), charge range 0–5, dynamic exclusion time 0.5 min, ion target intensity 10,000, ion intensity threshold 2500, and CID fragmentation energy 20–59 eV. (6) The database search software PaSER (version number: 2023) was used for mass spectrum file processing. The software’s TIMScore mode introduced the CCS dimension to provide higher PSM (peptide–spectrum match), peptide and protein identification numbers for 5D-proteomics, greatly increasing protein sequence coverage. In algorithms that do not support CCS values, only two dimensions of data can be used to evaluate FDR. In the TIMScore algorithm that supports CCS values, candidate peptides can evaluate FDR in three dimensions, which can improve the accuracy and precision of the results and help verify PSMs that previously scored poorly in traditional two-dimensional data. (7) Uniprot_Rattus_norvegicus_20230312_54764_10116 was selected as the protein comparison database in this project.

### 2.7. Bioinformatics Analysis

(1) We used Blast2GO (V1.4.4) to perform GO annotation on the target protein collection. The version number used in the GO database in this project is the following: go.obo (1 July 2019). (2) We used KOALA (KEGG Orthology And Links Annotation, V2.3) software to compare the KEGG GENES database, annotated the target protein set with KEGG pathways, classified the target protein sequence into KO categories, and automatically obtained the target protein based on the KO classification sequence pathway information involved. The version number used by the KEGG database in this project is KO_INFO_END.txt (24.03.2023). (3) The GO/KEGG pathway enrichment analysis algorithm was consistent and based on Fisher’s exact test, comparing the distribution of each GO entry or KEGG pathway in the target protein set and the overall protein set to evaluate a GO significance level of the item or KEGG pathway enrichment. (4) Domain annotation and enrichment analysis used the Interpro database to perform functional domain annotation analysis on differential proteins. We compared the distribution of the target protein in the total protein collection based on Fisher’s exact test to evaluate the significance level of the enrichment of a certain functional domain. (5) Subcellular localization analysis WoLF PSORT (https://wolfpsort.hgc.jp/) software (accessed on 16 March 2023) was used to predict the subcellular localization of proteins. (6) Cluster analysis normalized the quantitative information of the target protein set, and then used the matplotlib software (version number: V3.3.4) package in Python to classify simultaneously the two dimensions of sample and protein expression (distance algorithm: Euclidean; connection method: average linkage), and finally generated a hierarchical clustering heat map. (7) Transcription factor analysis used AnimalTFDB3.0 (the Animal Transcription Factor Database) for transcription factor prediction analysis. (8) Protein interaction network analysis used the String database (https://www.string-db.org/, accessed on 16 March 2023) (version number:V11.5) to obtain a list of proteins that interact directly or indirectly with the target protein through AnyChart software (V8.11.0.1934), a software which generates interaction network analysis results. (9) Phosphorylated conserved group sequence analysis used the phospho modifications information in the peptide data table to extract and organize the pre-aligned peptide set from the corresponding protein sequence (centered on the phosphorylation site, extending forward and backward by 6 amino acids), and then used Motif-X (meme-5.0.1) software to analyze the conserved motifs around the phosphorylated serine, threonine and tyrosine residues. GO and Kyoto Encyclopedia of Genes and Genomes (KEGG) analysis were used to further understand the signaling pathways involved in dapagliflozin promoting angiogenesis after hindlimb ischemia.

### 2.8. Cell Culture

Human umbilical vein endothelial cells (HUVECs) were purchased from the American Type Culture Collection (Manassas, VA, USA) and were cultivated in DMEM medium supplemented with 10% fetal bovine serum (Gibco, Grand Island, NY, USA) and 1% penicillin/streptomycin in an incubator (5% CO_2_, 95% O_2_). Note: ischemia and hypoxic conditions referred to as HUVECs were cultivated in DMEM medium without 10% fetal bovine serum (Gibco, Grand Island, NY, USA) and placed in an anoxic incubator (HF 212UV; Thermo Fisher Scientific Ltd., Waltham, MA, USA) with 5% CO_2_, 1% O_2_, and 94% N_2_ at 37 °C and were passaged regularly.

Before conducting subsequent cell experiments, we explored the appropriate dosage concentration of dapagliflozin for HUVECs. We set four dosage concentrations (1 μm, 5 μm, 10 μm, and 20 μm), and also established a blank control group and a DMSO control group (dapagliflozin was used for DMSO for dissolution), intervened the cells for 24 h respectively, and then cultured them under ischemic and hypoxic conditions for 48 h. Then, the cells in each group were subjected to a CCK-8 experiment to evaluate cell proliferation ability, a transwell experiment to evaluate cell migration ability, a Matrigel experiment to evaluate tubule formation ability, and a Western blot experiment to detect the protein expression of corresponding pro-angiogenic factors.

### 2.9. Ischemia/Hypoxia (I/H) Model and Treatments

For dapagliflozin treatment, cells were treated with the indicated doses of dapagliflozin dissolved in 10% dimethyl sulfoxide (DMSO) for 24 h. After dapagliflozin treatment, the culture medium was replaced with DMEM without fetal bovine serum (FBS), and the cells were placed under the above-mentioned hypoxic conditions for 48 h. The cells were divided into 4 groups: without any treatment (I/H group), the cells treated with dapagliflozin (5 μm, for 24 h, dapagliflozin group), the cells treated with dapagliflozin with LY294002 (dapagliflozin + LY294002 group, with LY294002 intervention given for 60 min before dapagliflozin intervention), and the cells treated with dapagliflozin with L-name (dapagliflozin + L-name group, with L-name intervention given for 60 min before dapagliflozin intervention); in addition, two control groups were added, namely the normal blood oxygenation group and the normal blood oxygenation plus dapagliflozin group.

### 2.10. CCK-8 Proliferation Ability Assay

Cell proliferation capacity was assessed by CCK-8 assay. Briefly, after the HUVECs received appropriate treatment and were seeded in 96-well plates, 10 μL CCK-8 (Dojindo Lab, Tokyo, Japan) solution was added to each well at a 1/10 dilution, followed by a further 2 h incubation. Absorbance was measured at 450 nm with a microplate reader (ELX 800; Bio-Tek, Winooski, VT, USA). The mean optical density of four wells in different groups was used to calculate the percentage of cell viability.

### 2.11. Transwell Migration Assay

Twenty-four-well Boyden transwell chambers (Corning, Cambridge, MA, USA) were employed to determine cell migration. In brief, the original incubation mediators (600 μL) were added to lower chambers, and corresponding pretreated HUVECs (1 × 10^5^/well/100 μL) were reseeded in upper chambers in serum-free medium. After 24 h, they were fixed with 4% paraformaldehyde and stained with crystal violet, and then the migrated HUVECs were counted using a light microscope (×10 magnification).

### 2.12. Matrigel Tubule Formation Assay

The HUVECs were detached by trypsinization and, after neutralization of trypsin, they were counted and resuspended. The HUVECs were seeded at 2 × 10^5^ cells per well in Matrigel (BD Biosciences, San Jose, CA, USA) pre-coated 96-well plates. Tube formation was quantified 6–8 h later. Digital images of endothelial tubes were obtained using a phase-contrast microscope (Leica, Wetzlar, Germany). Tube formations were measured blind by an independent observer, giving the total tube length per image.

### 2.13. Western Blotting Analysis (Cellular Protein)

Western blotting detected the expression of PI3K/Akt/eNOS signaling pathway proteins in HUVECs in each group. After extracting the total protein from cells, the concentration and purity were detected, electrophoresis was performed, and it was transferred to the membrane. It was blocked with skimmed milk powder at room temperature for 2 h, the primary antibodies (anti-eNOS, anti-p-eNOS (Ser1177), anti-Akt, anti-p-Akt (Ser473), anti-vascular endothelial growth factor A (VEGFA) (Beyotime, Haimen, China)) and internal reference (anti-GAPDH (Cell Signaling Technology, Danvers, MA, USA)) were added, respectively, and it was diluted at 1:1000. The next day, we added the secondary antibody, incubated it at room temperature for 1 h, washed the membrane, and performed chemiluminescence. Based on the gray value ratio of each protein band, the relative protein expression was calculated. Original western blots are available in the Supplementary Materials.

### 2.14. Statistical Analysis

Data were presented as mean ± SE. A two-way ANOVA was performed to evaluate the strain and condition factors. Tukey’s post hoc test was employed for multiple comparisons when a statistical significance was obtained with ANOVA. *p* values < 0.05 were considered significant.

## 3. Results

The three groups of rats were weighed at the beginning of the experiment, which is the day when the hindlimb ischemia model was established (d0), and at the end of the experiment, which was the 21st day after hindlimb ischemia surgery (d21), respectively, and the peripheral blood of the rats was collected for blood glucose measurement, liver and kidney function, serum sodium, blood potassium, glycated serum protein and other indicators (Table 1 and Table 2). At d0, we found there were no statistical differences in the weight, blood glucose, liver function, kidney function, blood sodium, and blood potassium indicators of the three groups of rats. At the end of the experiment, that is, on the 21st day after hindlimb ischemia surgery (d21), there was no statistical difference in the body weight of the dapagliflozin group and model group, compared with the control group. Meanwhile, there was a statistically significant difference between the dapagliflozin group and the model group, indicating that dapagliflozin has a weight-reducing effect on rats. The blood glucose and glycated serum protein on the same day d21 showed no statistical difference between the dapagliflozin group and control group, or between the model group and control group, while there was a statistically significant difference between the dapagliflozin group and model group, indicating that dapagliflozin has a lowering effect on the blood glucose of rats. There was no statistical difference in liver function and kidney function of the three groups of rats on day 21; also, there was no statistically significant difference between the dapagliflozin group and the model group, indicating that dapagliflozin did not damage liver and kidney function.

### 3.1. Dapagliflozin Increased Hindlimb Blood Perfusion and Vascular Density in the Hindlimb Ischemia Rat Model

**Figure 1:**
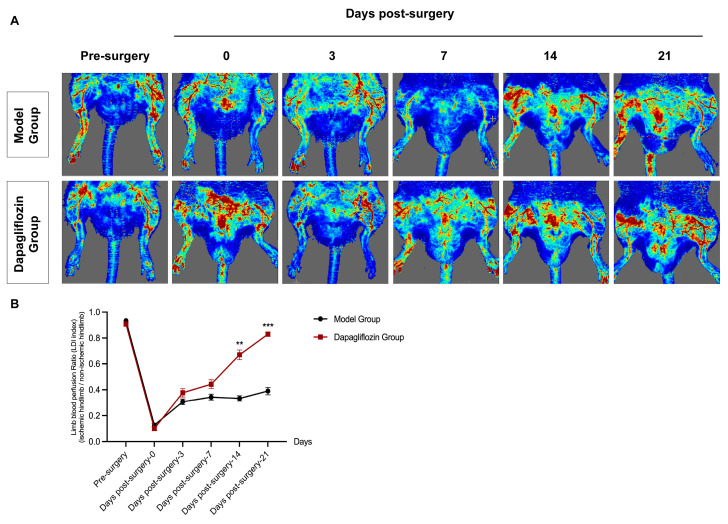
Effect of dapagliflozin on blood flow recovery after hindlimb ischemia. (**A**) Representative images of hindlimb laser doppler perfusion testing. (**B**) Line chart indicating limb perfusion ratio (Laser Doppler Image index). Data are expressed as mean ± standard error (*n* = 3). ** *p* < 0.01, *** *p* < 0.001 compared to model group.

Dapagliflozin can significantly increase regional blood flow in hindlimb ischemic rats. Figure 1A shows representative images of laser Doppler blood flow imaging of the bilateral hindlimbs of rats before surgery, after surgery, and on days 3, 7, 14, and 21 after surgery. The ratio of the blood flow perfusion of the ischemic left hindlimb to the healthy right hindlimb was recorded for statistical analysis. The statistical results showed that the blood flow perfusion of the affected hindlimb in the two groups (model group and dapagliflozin group) decreased at the same level after surgery. On day 3, blood perfusion recovery began to occur in both groups, but no significant difference was found between the groups; on the 7th and 14th days after surgery, the recovery level of blood flow in the hindlimbs of rats in the dapagliflozin treatment group was better than that in the model group. On the 21st day after surgery, the blood perfusion of the ischemic hindlimbs of rats in the dapagliflozin group was significantly improved compared with the model group. Compared with the model group (left femoral artery resection, receiving intragastric feeding with citric acid–sodium citrate buffer solution (1 mg/kg/d) for 21 consecutive days), which achieved 30% blood perfusion recovery on the 21st day after surgery, the blood perfusion of the ischemic hind limbs of the rats in the dapagliflozin group recovered by 80% (Figure 1A,B).

**Figure 2:**
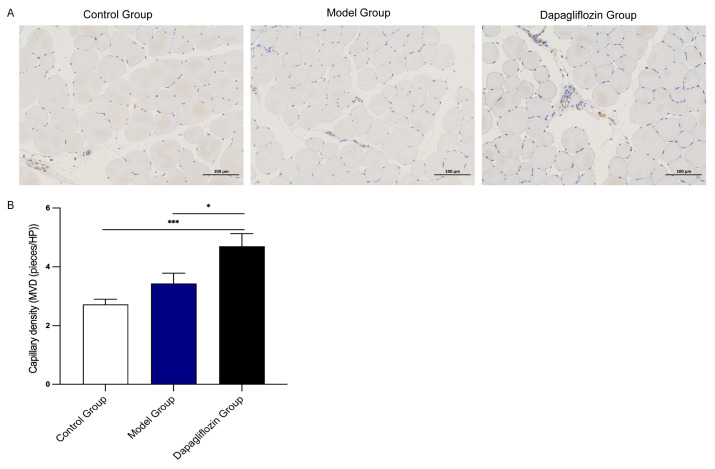
Effect of dapagliflozin treatment on capillary density of hindlimbs after hindlimb ischemia. (**A**) Schematic diagram of CD31 immunohistochemical staining of hindlimb muscles on day 21 after ischemic intervention. (**B**) Statistical chart of quantitative analysis of CD31-positive blood vessels. Data are expressed as mean ± standard error (*n* = 10). * *p* < 0.05, *** *p* < 0.001. Scale bar indicates 100 μm.

Quantitative analysis of CD31-positive blood vessels found that compared with the control group (2.72 ± 0.18), the blood vessel density of the ischemic hindlimbs of rats in the model group and dapagliflozin-treated group increased after 3 weeks, and the model group (3.44 ± 0.35), the dapagliflozin treatment group (4.70 ± 0.44), and the blood vessel density of the dapagliflozin treatment group was also significantly higher than that of the model group, showing the highest blood vessel density. There were statistical differences between the dapagliflozin group and the model group, and also between the dapagliflozin group and the control group. The results show that dapagliflozin can induce the formation of new blood vessels, and treatment with dapagliflozin for 21 days can promote angiogenesis after ischemia in the hindlimbs of rats (Figure 2).

### 3.2. Phosphoproteomics Test Results: PI3K-Akt-eNOS Signaling Pathway Was Closely Related to the Effect of Dapagliflozin on Post-Ischemic Angiogenesis

**Figure 3:**
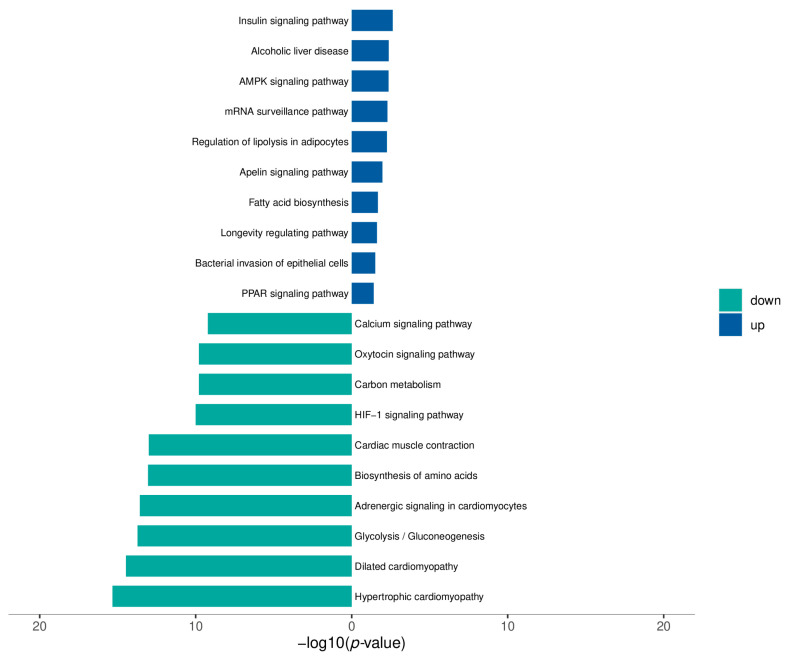
KEGG pathway database-determined gene enrichment results.

Phosphorylation is an important post-translational protein modification, and it is estimated that more than one-third of all proteins in mammalian cells can be phosphorylated. Phosphorylated proteins play important roles in cellular functions [19,20,21,22,23], including signal transduction, differentiation, proliferation, cycle regulation, metabolism, transcription and translation, degradation, and survival. Phosphoproteomic analyses identify, catalog, and characterize phosphorylated proteins. Because phosphorylated proteins are involved in numerous biological processes, the current study aimed to further elucidate the mechanism of dapagliflozin affecting angiogenesis after hindlimb ischemia by detecting phosphorylated proteins, to clarify the effect of dapagliflozin on angiogenesis after hindlimb ischemia, and to verify its mechanism of action at the cellular level. Three ischemic hindlimb adductor muscle tissue samples from rats in the model group and dapagliflozin group were taken for phosphoproteomic testing. The results showed that using the difference fold (fold-change >± 1.5 times) and *p* value (*p* < 0.05) as the criteria for screening differential proteins, a total of approximately 376 differentially expressed phosphorylated proteins were screened out. From these differentially expressed phosphorylated proteins, we found that Akt was significantly changed. Compared with the model group, Akt was up-regulated in the dapagliflozin administration group. In combination with previous related studies, it was shown that phosphorylated Akt is associated with post-ischemic angiogenesis [24,25]. KEGG pathway enrichment analysis was used to study the different pathways involved in dapagliflozin affecting angiogenesis after hindlimb ischemia (Figure 3). The main signaling pathway involved in dapagliflozin affecting post-ischemic angiogenesis was the AMPK pathway. Akt plays a very important regulatory role in the AMPK signaling pathway. We can see that it has PI3K upstream and eNOS downstream. In our previous studies, we confirmed that PI3K-Akt-eNOS is closely related to angiogenesis. Our proteomic results also confirmed this, and we will next verify this mechanism at the cellular level [26,27].

### 3.3. Mechanism of Dapagliflozin in the Hindlimb Ischemia Rat Model

**Figure 4:**
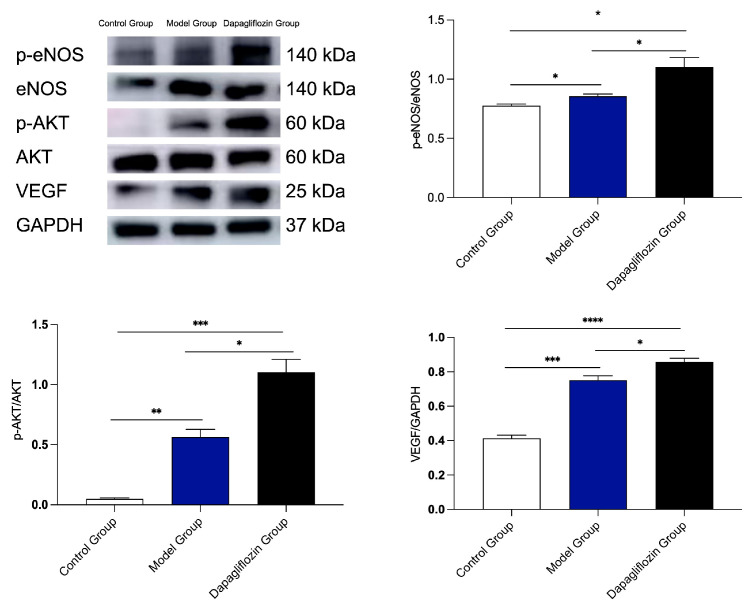
Effect of dapagliflozin on the expression of pro-angiogenic factors p-eNOS, p-Akt, eNOS, Akt and VEGF protein in ischemic hindlimbs. On the 21st day after hindlimb ischemia surgery, Western blot was used to detect the contents of p-eNOS, p-Akt, eNOS, Akt and VEGF in ischemic muscle tissue to evaluate the pro-angiogenic effect. Data are expressed as mean ± standard error, * *p* < 0.05, ** *p* < 0.01, *** *p* < 0.001, **** *p* < 0.0001.

In order to further explore the possible mechanism of angiogenesis promoted by dapagliflozin, based on the phosphoproteomic detection results, we evaluated the expression of pro-angiogenic factors p-Akt, p-eNOS, total-Akt, total-eNOS and VEGF protein in ischemic muscle tissue of the three groups (control group, model group, and dapagliflozin group), on the 21st day after femoral artery ligation. The results showed that dapagliflozin could increase the expression of phosphorylated protein Akt, eNOS, and VEGF. Compared with the control group, the phosphorylated pro-angiogenic factors were increased in both the model group and the dapagliflozin group, and the dapagliflozin-treatment group had the highest expression of phosphorylated pro-angiogenic factors among the three groups. There were statistical differences between the dapagliflozin group and control group, and between the dapagliflozin group and model group (Figure 4). The dapagliflozin administration allowed for better angiogenic factor expression than the other group.

### 3.4. Selection of Drug Concentration for Dapagliflozin to Interfere with HUVECs

**Figure 5:**
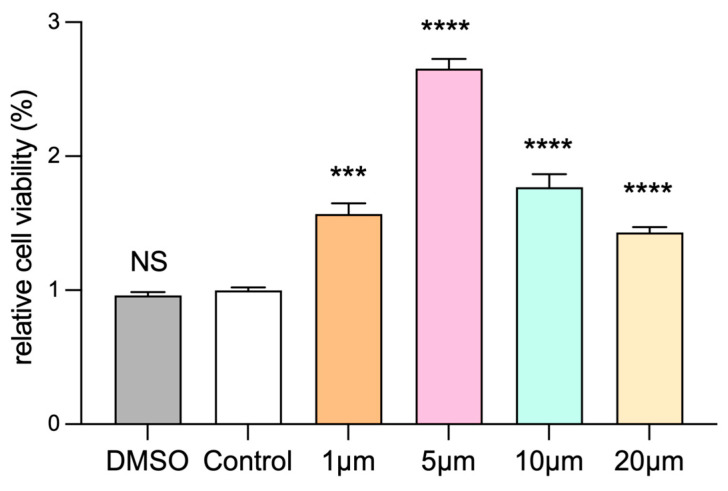
Effects of different concentrations of dapagliflozin interfering with HUVECs on cell proliferation. Data are expressed as mean ± standard error (*n* = 5). *** *p* < 0.001, **** *p* < 0.0001 vs. control group; NS means “not statistical” vs. control group.

**Figure 6:**
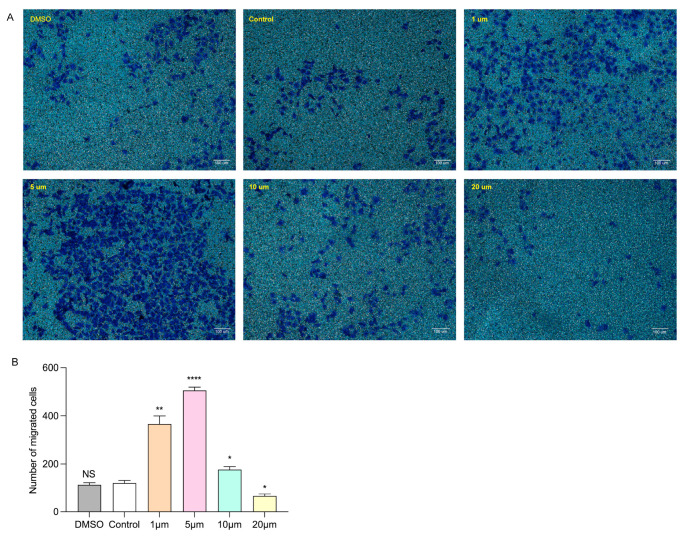
Effects of different concentrations of dapagliflozin interfering with HUVECs on cell migration ability. (**A**) Transwell diagram. (**B**) Transwell analysis diagram. Data are expressed as mean ± SE (*n* = 3). * *p* < 0.05, ** *p* < 0.01, **** *p* < 0.0001 vs. control group; NS means “not statistically” vs. control group. Scale bar indicates 100 μm.

**Figure 7:**
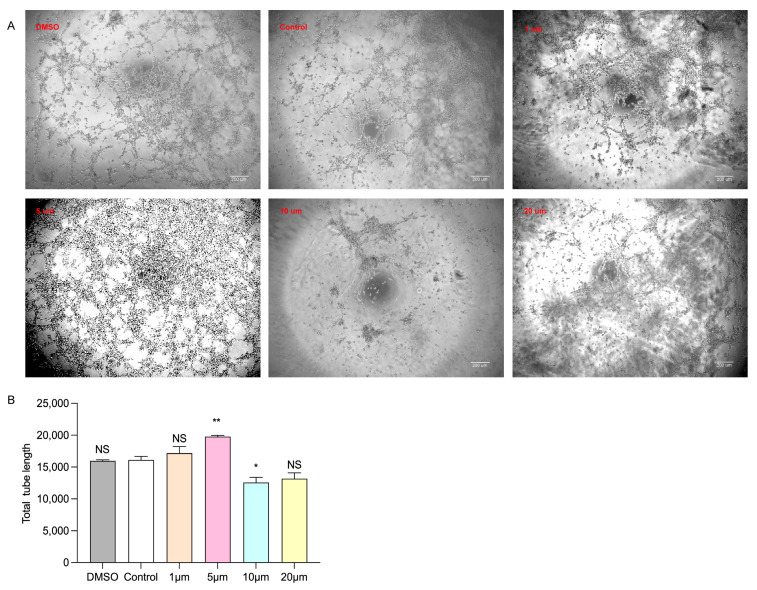
Effects of different concentrations of dapagliflozin interfering with HUVECs on their tubule formation ability. (**A**) Tube-generation schematic diagram. (**B**) Tube-generation analysis diagram. Data expressed as mean ± SE. * *p* < 0.05, ** *p* < 0.01 vs. control group; NS means “not statistically” vs. control group. Scale bar indicates 200 μm.

**Figure 8:**
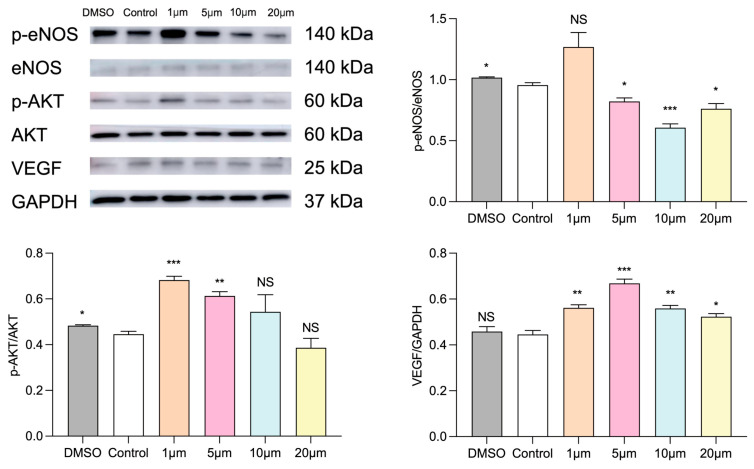
Expression of angiogenic factors after intervention of different concentrations of dapagliflozin in HUVECs. Western blot schematic diagram and band quantitative analysis diagram. * *p* < 0.05, ** *p* < 0.01, *** *p* < 0.001 vs. control group; NS means “not statistically” vs. control group.

In order to explore the appropriate concentration of dapagliflozin to intervene in HUVECs, we first administered different concentration gradients of dapagliflozin to HUVECs under ischemic and hypoxic conditions before starting the cell experiment. The set concentration gradients were 1 μm, 5 μm, 10 μm and 20 μm. In addition, because the dapagliflozin reagent used to interfere with cells was dissolved in DMSO, we set up a DMSO control group and a blank control group. The cells were intervened for 24 h and then cultured under ischemic and hypoxic conditions for 48 h. Then, the cells in each group were subjected to the CCK-8 experiment to evaluate cell viability, the transwell experiment to evaluate cell migration ability, the Matrigel experiment to evaluate tubule formation ability, and the Western blot experiment to detect the protein expression levels of corresponding pro-angiogenic factors. From the results of CCK-8, we can see that there was no statistical difference in the cell proliferation ability of HUVECs between the DMSO group and the control group. The cell proliferation ability was linearly related to the concentration of dapagliflozin. The cell viability was optimal at the intervention concentration of 5 μm. At the intervention concentration of 10 μm, the cell viability began to decline, but it was still higher than the control group. At the intervention concentration of 20 μm, the cell viability continued to decline, but it was still higher than the control group (Figure 5). We then used transwell experiments to evaluate the changes in cell migration ability after intervening the HUVECs with different concentrations of dapagliflozin under ischemic and hypoxic conditions. It can be seen from the results that there was no statistical difference in the cell migration ability of HUVECs between the control group and DMSO group. The cell migration ability was linearly related to the concentration of dapagliflozin. The cell migration ability was the best at the intervention concentration of 5 μm. At the intervention concentration of 10 μm, the cell migration ability began to decrease, but it was still higher than the control group. At the intervention concentration of 20 μm, the cell migration ability decreased significantly (Figure 6). The tubule formation assay was used to evaluate the changes in the tubule formation ability of HUVECs after different concentrations of dapagliflozin intervened under ischemic and hypoxic conditions. We observed that after ischemia and hypoxic injury, at the same time, the tubule formation of HUVECs in the control group was weakened. As shown in Figure 7, the cells aggregated into clusters or were broken, and the lumen-like structure was significantly less than that of those cultured in complete medium under normoxic conditions. The DMSO group and the control group had similar tubule formation. The 1 μm group was slightly better, but there was no significant statistical difference compared with the control group. The 5 μm group had significantly enhanced tubule formation ability and increased lumen-like structures. The ability of tubule formation in the 10 μm and 20 μm groups decreased significantly (Figure 7). In order to further explore the expression of angiogenic factors after different concentrations of dapagliflozin intervening in HUVECs under ischemic and hypoxic conditions, we extracted the cellular proteins of each group and performed Western blot detection. It can be seen from the experimental results that there was no significant difference in the expression levels of eNOS and Akt total protein of cells in each group; the expression level of p-eNOS was the highest in the 1 μm group; the expression level of p-Akt was higher in both the 1 μm and 5 μm groups. VEGF plays a crucial role in angiogenesis, so we also measured the expression level of VEGF. The results showed that the expression levels of VEGF in the 1 μm group and the 5 μm group were both higher than those in the control group, and both were statistically significant differences compared with the control group (Figure 8). Taken together, these results showed that treatment with 1 μm and 5 μm dapagliflozin for 24 h can significantly increase cell viability in a dose-dependent manner, while the 10 μM- and 20 μM-dose groups have no significant difference from the control group. Treatment with high-concentration dapagliflozin had no obvious effect on cells, so we subsequently used 5 μM dapagliflozin for further cell experiments.

### 3.5. Dapagliflozin Facilitated the HUVEC Proliferation In Vitro

**Figure 9:**
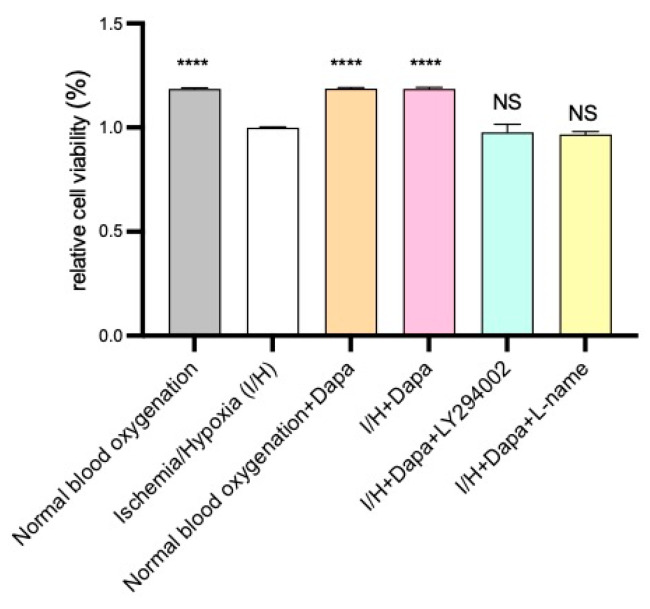
Effect of dapagliflozin on HUVEC proliferation. Compared with the normal blood and oxygen-supply group, the cell proliferation ability of the ischemic and hypoxic group was significantly reduced. The cell proliferation ability of ischemic and hypoxic cells recovered well after dapagliflozin intervention. Dapagliflozin intervention promoted the proliferation of HUVECs. This effect can be blocked by L-name or LY294002. Data are expressed as mean ± SE, **** *p* < 0.0001 vs. I/H group; NS means “not statistically” vs. I/H group.

The CCK-8 assay was performed to evaluate HUVEC proliferation ability in response to dapagliflozin administration. In the present study, we found that the dapagliflozin administration (5 μm) facilitated the HUVEC proliferation better than the control group under ischemic and hypoxic conditions. Moreover, this benefit could be markedly attenuated using eNOS inhibitor L-name or PI3K inhibitor LY-294002 (Figure 9).

### 3.6. Dapagliflozin Enhanced the HUVEC Migration In Vitro

**Figure 10:**
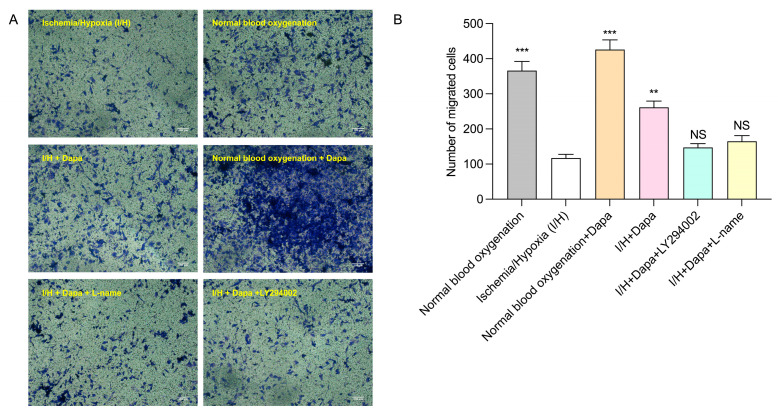
Effect of dapagliflozin intervention on HUVEC migration. Migrated cells were stained (**A**) and quantitative analysis of migrated cells was carried out (**B**). Values are mean ± SE, ** *p* < 0.01, *** *p* < 0.001 vs. I/H group; NS means “not statistically” vs. I/H group. Scale bar indicates 100 μm.

The transwell migration assay was performed to evaluate the HUVEC migration ability in response to dapagliflozin administration. In the present study, we found that the dapagliflozin administration (5 μm) group showed more migrated HUVECs than the control group under ischemic and hypoxic conditions. Moreover, this benefit could be markedly attenuated using eNOS inhibitor L-name or PI3K inhibitor LY-294002 (Figure 10).

### 3.7. Dapagliflozin Augmented the HUVEC Tube Formation In Vitro

**Figure 11:**
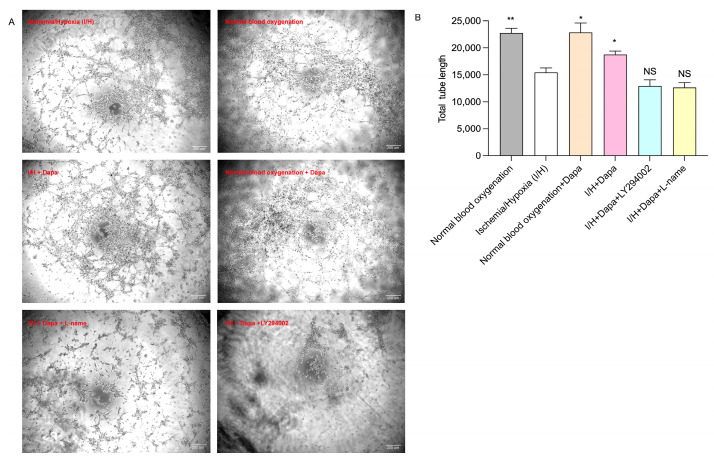
Tube formation ability was evaluated by Matrigel assay. (**A**) Representative images of tube formation in HUVECs. (**B**) Quantitative analysis of total tube length. Values are mean ± SE, * *p* < 0.05, ** *p* < 0.01 vs. I/H group; NS means “not statistically” vs. I/H group. Scale bar indicates 200 μm.

In the tube formation assay, dapagliflozin administration (5 μm) increased the tubule formation induced by I/H in HUVECs. Moreover, this benefit could be markedly attenuated using eNOS inhibitor L-name or PI3K inhibitor LY-294002 (Figure 11).

### 3.8. Dapagliflozin Enhanced the Expression of Pro-Angiogenic Factors In Vitro, and This Effect Could Be Blocked by PI3K Inhibitors or eNOS Inhibitors, Proving That Dapagliflozin Upregulated VEGF Expression by Activating the PI3K-Akt-eNOS Signaling Pathway, Thereby Promoting Post-Ischemic Angiogenesis

**Figure 12:**
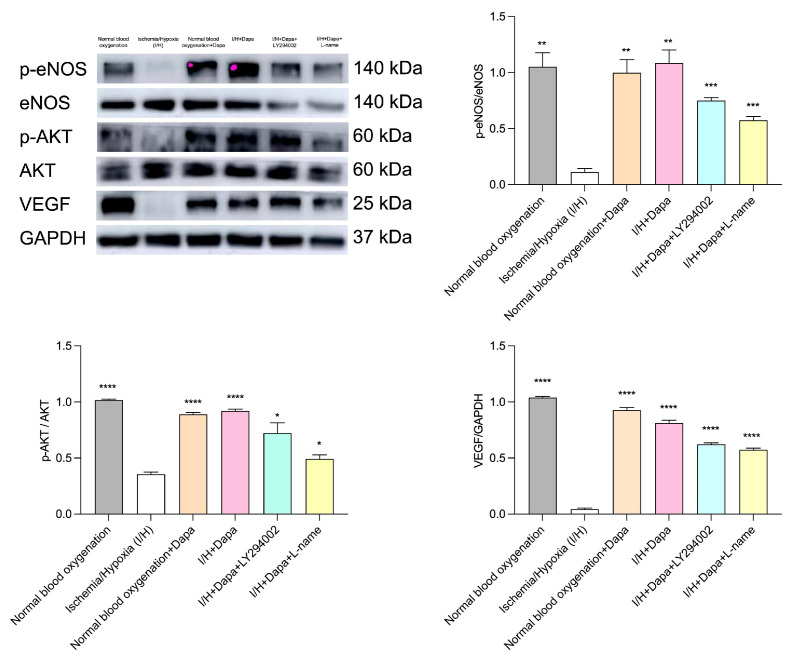
Effect of dapagliflozin intervention on the expression of pro-angiogenic factors. Dapagliflozin intervention caused the upregulation of p-eNOS, p-AKT and VEGF in HUVECs in I/H. L-name or LY294002 can block this effect. Data are expressed as mean ± standard error, * *p* < 0.05, ** *p* < 0.01, *** *p* < 0.001, **** *p* < 0.0001 vs. I/H group; NS means “not statistical” vs. I/H group.

To clarify the proangiogenic effects of dapagliflozin on eNOS, Akt and VEGF activities in ischemic/hypoxia-treated HUVECs, we assessed the expression of eNOS, Akt and VEGF in treated HUVECs. As illustrated in Figure 12, the administration of dapagliflozin upregulated phosphorylation of eNOS and phosphorylation of Akt and VEGF in ischemic/hypoxia-treated HUVECs. The benefits can be neutralized by the administration of L-name andLY-294002 (Figure 12).

## 4. Discussion

Dapagliflozin is a sodium glucose transporter 2 inhibitor (SGLT2i), a new type of hypoglycemic drug that can inhibit the absorption of glucose and sodium by the kidney [5]. In 1835, C, Petersen extracted “Phlorizin” from the apple root bark. Since then, the first non-selective SGLT1 inhibitor has been born. Its main pharmacological effect is to block the reabsorption of glucose by SGLT in renal tubules and the small intestine. In the 1870s, people found in continuous research that “phlorizin” non-selectively inhibits SGLT1 in small intestinal epithelial cells, causing “phlorizin” to have serious adverse reactions such as osmotic diarrhea, indigestion, etc., and it was found that the utilization rate of “barktin”, which was produced after hydrolase in the intestinal cavity decomposes “phloridzin”, is extremely low, so it is not widely used in clinical treatment [28]. After more than 150 years of continuous innovation in science and technology, SGLT2 inhibitors have finally passed phase III clinical trials and are on the market. Compared with SGLT1 inhibitors, SGLT2 inhibitors have stronger specificity and selectivity, and a higher ability to transport glucose. And the sites of action are almost all in the kidneys. These include dapagliflozin, empagliflozin and canagliflozin, as well as the SGLT1/2 dual-channel inhibitor soxagliflozin. Recent studies have shown that dapagliflozin has many effects that are independent of the hypoglycemic effect. Different SGLT2 inhibitors had different effects on different target organs. Some SGLT2 inhibitors could promote angiogenesis in target organs, and some SGLT2 inhibitors could inhibit angiogenesis in target organs. Our study mainly studied the effect of oral administration of dapagliflozin on angiogenesis after hindlimb ischemia, and its mechanism of action. First, we observed at the animal level the effect of dapagliflozin on post-ischemic neovascularization in the hindlimbs of rats after ischemia. It could be seen that 21 days after dapagliflozin administration, the blood perfusion of the ischemic hindlimbs of rats recovered by about 80%, and at the same time the capillary density of the ischemic hindlimbs of rats in the dapagliflozin group increased significantly, indicating that dapagliflozin could promote post-ischemic angiogenesis. In the animal experiment, because we used intragastric administration, we needed to observe the liver and kidney side effects of the drug. From Table 1 and Table 2, we drew the following conclusions: there was no statistical difference in liver function and kidney function of the three groups of rats on day 21, and also there was no statistically significant difference between the dapagliflozin group and the model group, indicating that dapagliflozin did not damage liver and kidney function. Currently, there are many studies on the mechanism of action of dapagliflozin, including anti-inflammation, anti-fibrosis, reduction of apoptosis, expression of angiogenic factors, etc. The study by Elkazzaz, S.K. et al. had shown that compared with the diabetic nephropathy-rat group, the histological examination, inflammation and apoptosis markers in the dapagliflozin-treatment group were dose-dependently improved, and the expression of the angiogenic factor VEGF was improved [24]. Additionally, there were studies demonstrating a modulatory role of dapagliflozin in endothelial dysfunction. Research by Zhang, W. et al. showed that dapagliflozin-loaded exosome mimic could promoting diabetic wound healing through HIF-1α-mediated enhancement of angiogenesis [25]. Vascular endothelial injury was the initial factor in the occurrence of peripheral arterial disease. Our study had confirmed that dapagliflozin could protect the function of vascular endothelial cells, but through which signaling pathway does dapagliflozin promote angiogenesis after hindlimb ischemia? Therefore, we performed phosphoproteomics detection on the ischemic left-hindlimb adductor muscle tissue of rats in the model group and dapagliflozin group. Phosphorylation is an important post-translational protein modification, and it is estimated that more than one-third of all proteins in mammalian cells can be phosphorylated. Phosphorylated proteins play important roles in cellular functions [19,20,21,22,23], including signal transduction, differentiation, proliferation, cycle regulation, metabolism, transcription and translation, degradation, and survival. Phosphoproteomic analyses identify, catalog, and characterize phosphorylated proteins [29]. Because phosphorylated proteins are involved in numerous biological processes, in our study phosphorylproteomics was used to further elucidate the possible signaling pathway of dapagliflozin in promoting angiogenesis after hindlimb ischemia.

Based on the expression of differentially phosphorylated proteins, we selected the PI3K-Akt-eNOS signaling pathway, which was involved in the regulation of multiple cell functions, such as proliferation, differentiation, apoptosis and glucose transport. We then verified this mechanism directly at the endothelial cell level.

PI3K-AKT-eNOS was an important signaling pathway in the human body, involved in a variety of physiological and pathological processes, including cell adhesion, growth, survival, differentiation, and protein synthesis [30]. PI3K, epidermal growth factor (EGF), fibroblast growth factor (FGF), insulin-like growth factor 1 (IGF-1), calmodulin (CaM) and other growth factors could activate the PI3K pathway [31]. This pathway was not only involved in the occurrence and development of tumors, but also played an important role in normal tissue cells. The PI3K pathway played a crucial role in the formation of normal blood vessels during development. Mutations in the p110α catalytic subunit of PI3K led to severe vascular defects during embryonic development, indicating that PI3K was critical for endothelial cell migration and angiogenesis. The same studies had shown that the p110α subunit also regulated endothelial cell migration, barrier function, and junctional morphology [32]. The PI3K pathway was also involved in angiogenesis by regulating NO signaling in endothelial cells. Studies using eNOS knockout mice had shown that eNOS played a key role in VEGF-induced angiogenesis and vascular permeability, while earlier reports found that VEGF could induce NO production, which was attenuated by PI3K inhibitors. Generally [31], this regulation could occur through eNOS phosphorylation by AKT at serine 1177 residue [33]. This phosphorylation was required for VEGF-induced endothelial cell migration [34]. VEGF was known to be an effective angiogenic factor. VEGF could improve the vasoconstrictive function of ischemic tissue by directly stimulating the proliferation and migration of endothelial cells in the body, focusing on increasing circulation [35]. VEGF also stimulated endothelial cells to migrate these cells and form capillary-like structures in a PI3K-dependent manner [36]. Hypoxia could also enhance the phosphorylation of eNOS through the binding of HSP9O to eNOS and the activation of the PI3K pathway [37].

Our previous studies had shown that compared with normoxic HUVECs, ischemic-hypoxic HUVECs exhibited enhanced migration and angiogenesis abilities at early stages in vitro, but prolonged ischemia–hypoxia reduced endothelial cell tubular length and HUVECs migration ability. This experiment also evaluated the effect of ischemia and hypoxia on HUVECs, and observed that exposing HUVECs to ischemia and hypoxia for 48 h resulted in a significant reduction in the proliferation, migration, and tube-formation abilities of HUVECs in vitro. Ischemia and hypoxia caused huge damage to cells (Figure 9, Figure 10 and Figure 11). Furthermore, ischemia–hypoxia reduced p-Akt, p-eNOS, and VEGF levels in HUVECs (Figure 12). Under ischemic and hypoxic conditions, after dapagliflozin (5 μm) was administered to HUVECs, the cell proliferation, migration, and tubule formation abilities were significantly restored. Then, we testified the dapagliflozin administration promoting ischemic angiogenesis through enhancing endothelial proliferation, tube formation and migration via acting on the PI3K-Akt-eNOS-VEGF pathway of endothelial cells under ischemic and hypoxic conditions. We evaluated the expression of eNOS, Akt, and VEGF in treated HUVECs. As shown in Figure 12, administration of dapagliflozin up-regulated phosphorylation of eNOS Ser1177, phosphorylation of Akt Ser473, and VEGF in ischemia/hypoxia-treated HUVECs. These benefits could be counteracted by using L-name and LY-294002. The results indicated that dapagliflozin promotes post-ischemic angiogenesis by activating the PI3K-Akt-eNOS signaling pathway and up-regulating VEGF expression.

In summary, dapagliflozin could promote angiogenesis in rats after hindlimb ischemia. Dapagliflozin played a protective role in vascular endothelial function damage. Dapagliflozin could promote angiogenesis after ischemia. This effect might be achieved by promoting the activation of the PI3K-Akt-eNOS signaling pathway. This study provided a new perspective, new ideas, and a theoretical basis for the treatment of peripheral arterial disease. This study also had many limitations, such as the fact that there were other pathways and mechanisms by which dapagliflozin promoted post-ischemic angiogenesis. We had not yet explored this part, and other mechanisms related to angiogenesis (such as the anti-inflammatory and anti-fibrosis effect) might also help dapagliflozin to promote post-ischemic angiogenesis, which needs further study in the future.

## Figures and Tables

**Table 1:** Baseline characteristics of the control-group, model-group and dapagliflozin-group rats (d0).

Characteristics (Mean ± SE)	Control-Group (*n* = 10)	Model-Group (*n* = 10)	Dapagliflozin-Group (*n* = 10)	P (Model vs. Control-Group)	P (Dapa vs. Control-Group)	P (Dapa vs. Model-Group)
Weight (g)	265.80 ± 4.51	264.80 ± 5.02	264.60 ± 4.43	0.88	0.85	0.98
Glucose (mmol/L)	13.33 ± 0.24	13.29 ± 0.25	13.11 ± 0.20	0.92	0.49	0.57
GPT (U/L)	56.87 ± 3.69	58.86 ± 5.05	56.42 ± 2.81	0.75	0.92	0.68
GOT (U/L)	150.40 ± 25.90	142.50 ± 24.22	131.70 ± 8.43	0.83	0.50	0.68
Serum creatinine (umol/L)	33.80 ± 1.40	33.92 ± 1.35	31.18 ± 0.84	0.95	0.13	0.10
Blood urea nitrogen (mg/dL)	15.50 ± 0.98	14.85 ± 1.07	15.54 ± 1.12	0.66	0.98	0.66
Serum sodium (mmol/L)	151.00 ± 3.96	151.30 ± 3.12	153.60 ± 1.27	0.95	0.54	0.50
Serum potassium (mmol/L)	5.89 ± 0.22	5.75 ± 0.15	5.91 ± 0.15	0.60	0.95	0.46

Note: Data are shown as mean ± SE. Abbreviations: GPT, glutamic pyruvic transaminase; GOT, glutamic oxaloacetic transaminase; Dapa, dapagliflozin.

**Table 2:** The basic indexes of control-group, model-group and dapagliflozin-group rats (d21).

Characteristics (Mean ± SE)	Control-Group (*n* = 10)	Model-Group (*n* = 10)	Dapagliflozin-Group (*n* = 10)	P (Model vs. Control-Group)	P (Dapa vs. Control-Group)	P (Dapa vs. Model-Group)
Weight (g)	415.60 ± 13.32	402.50 ± 7.52	362.60 ± 10.10	0.40	0.01	0.01
Glucose (mmol/L)	12.27 ± 0.51	12.26 ± 0.58	10.52 ± 0.48	0.99	0.02	0.03
GPT (U/L)	56.22 ± 3.92	51.67 ± 4.57	49.93 ± 3.65	0.46	0.26	0.77
GOT (U/L)	133.20 ± 27.09	132.00 ± 25.41	119.30 ± 9.73	0.98	0.64	0.65
Serum creatinine (umol/L)	35.99 ± 1.00	35.23 ± 1.32	32.68 ± 1.10	0.65	0.04	0.16
Blood urea nitrogen (mg/dL)	15.79 ± 1.02	14.56 ± 1.12	13.98 ± 1.05	0.43	0.23	0.71
GSP (mmol/L)	1.47 ± 0.09	1.52 ± 0.10	1.15 ± 0.08	0.71	0.01	0.01

Note: Data are shown as mean ± SE. Abbreviations: GPT, glutamic pyruvic transaminase; GOT, glutamic oxaloacetic transaminase; GSP, glycated serum protein; Dapa, dapagliflozin.

## Data Availability

The datasets presented in the current study are available from the corresponding author on reasonable request.

## Supplementary Materials

The following supporting information can be downloaded at: https://www.mdpi.com/2218-273X/14/5/592/s1.
