# Supplementary figures and images for: Dapagliflozin Improves Angiogenesis after Hindlimb Ischemia through the PI3K-Akt-eNOS Pathway
Source: Biomolecules. 2024 May 16;14(5):592. doi: 10.3390/biom14050592 (PMC11487428; doi:10.3390/biom14050592)

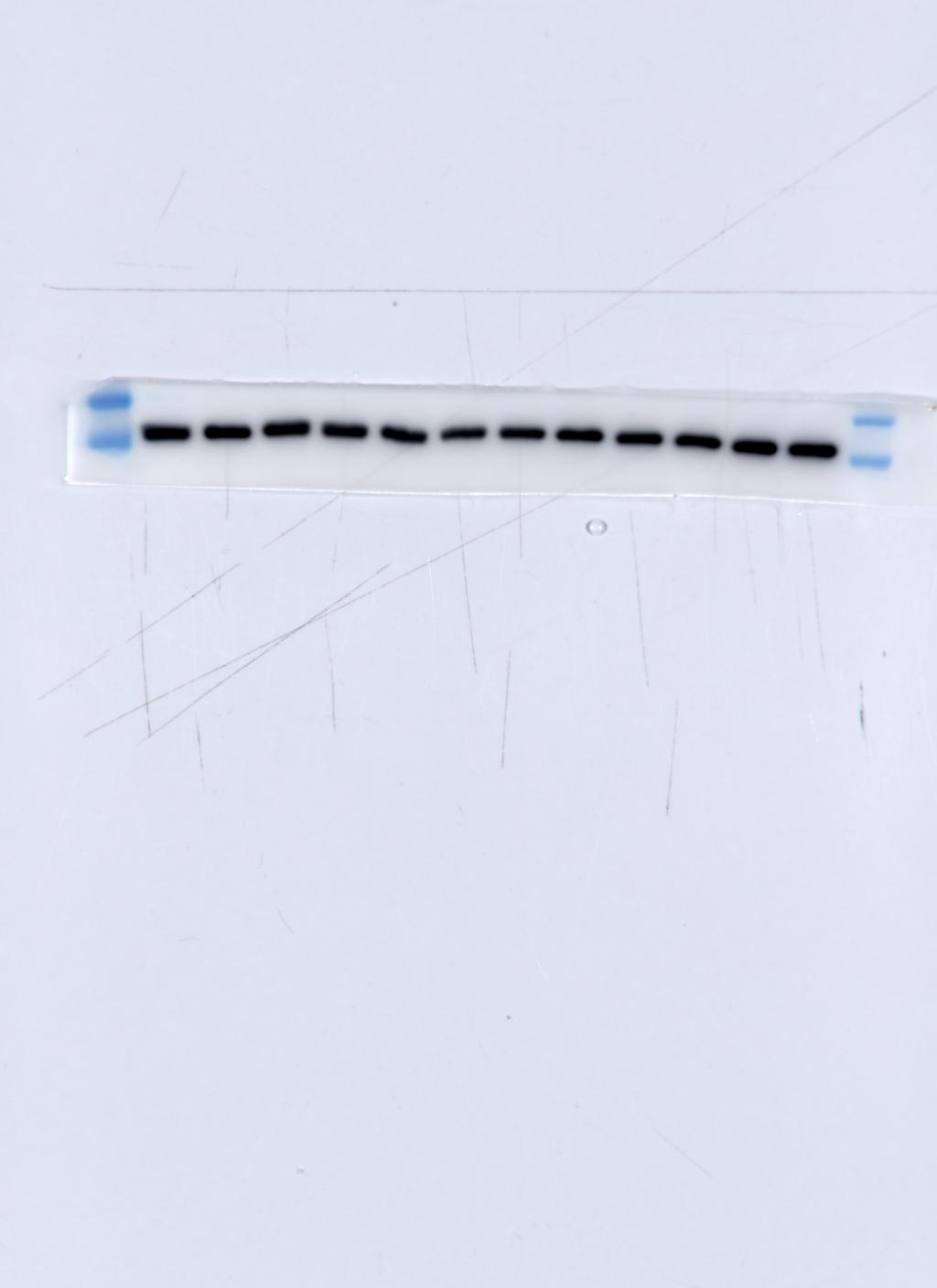

Supplement: Supplementary file 1 [file biomolecules-14-00592-s001.zip › Animal bands/HL gapdh/gapdh.jpg]

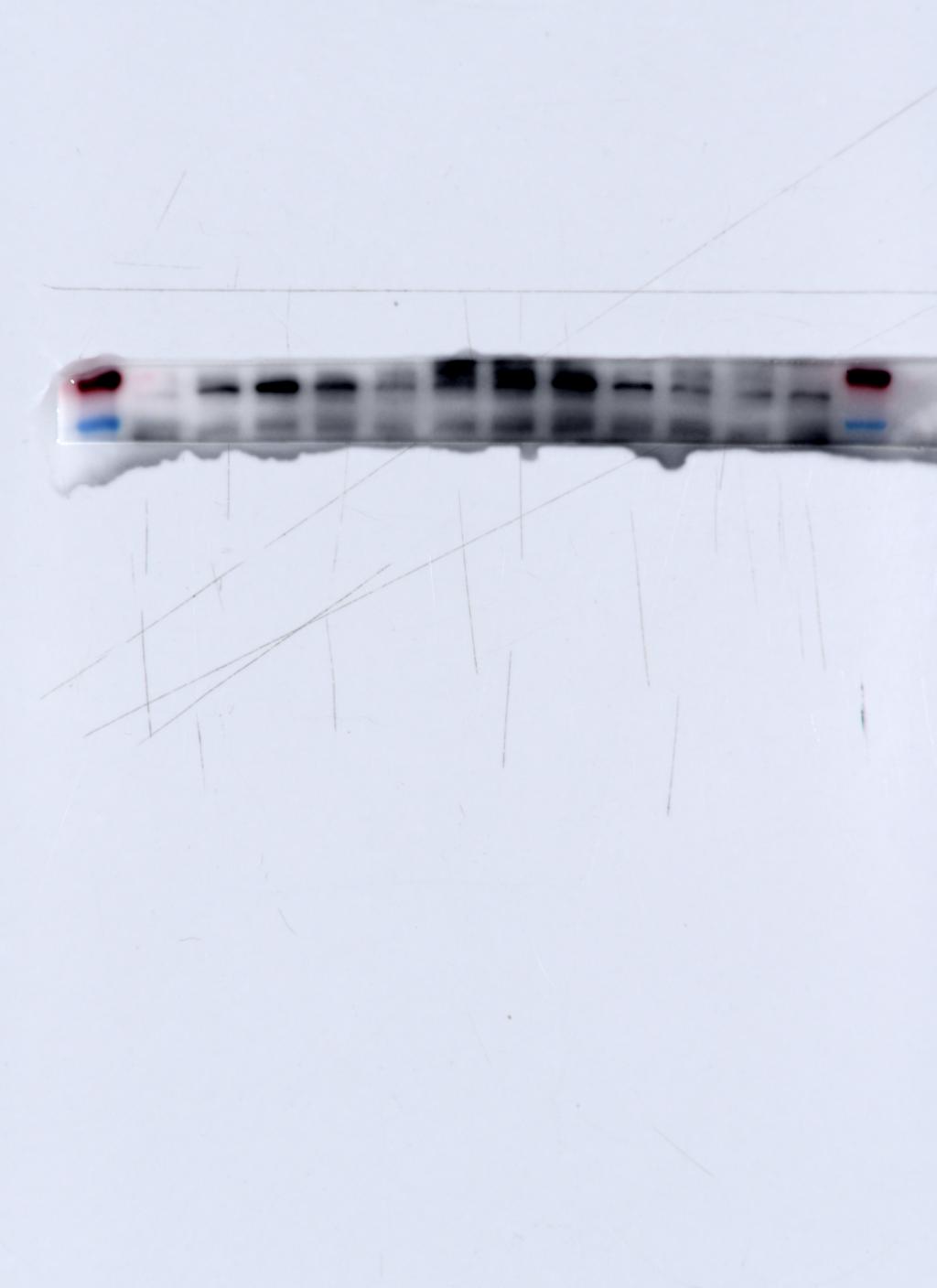

Supplement: Supplementary file 1 [file biomolecules-14-00592-s001.zip › Animal bands/HL p-akt/p-akt.jpg]

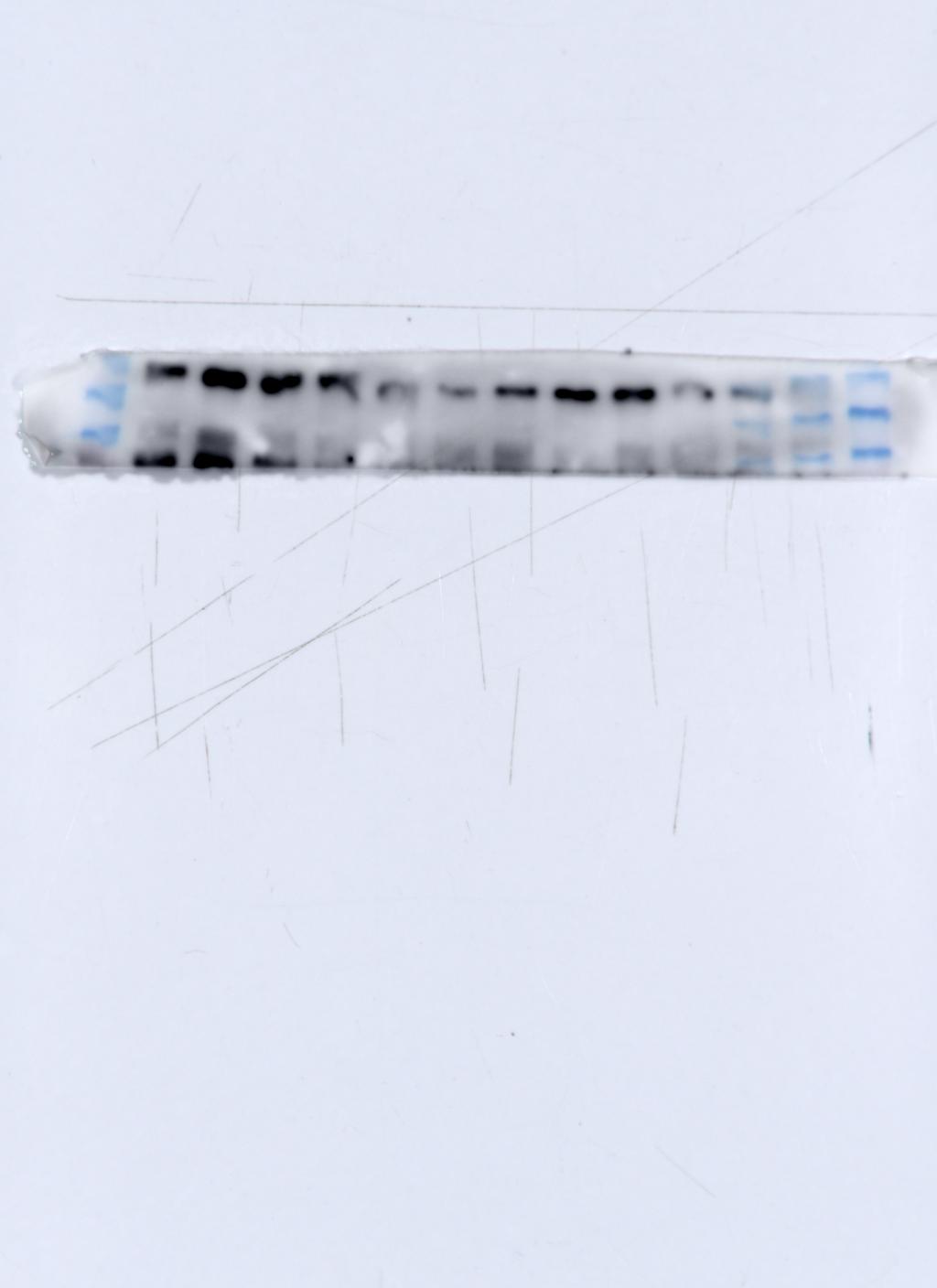

Supplement: Supplementary file 1 [file biomolecules-14-00592-s001.zip › Animal bands/HL enos/enos.jpg]

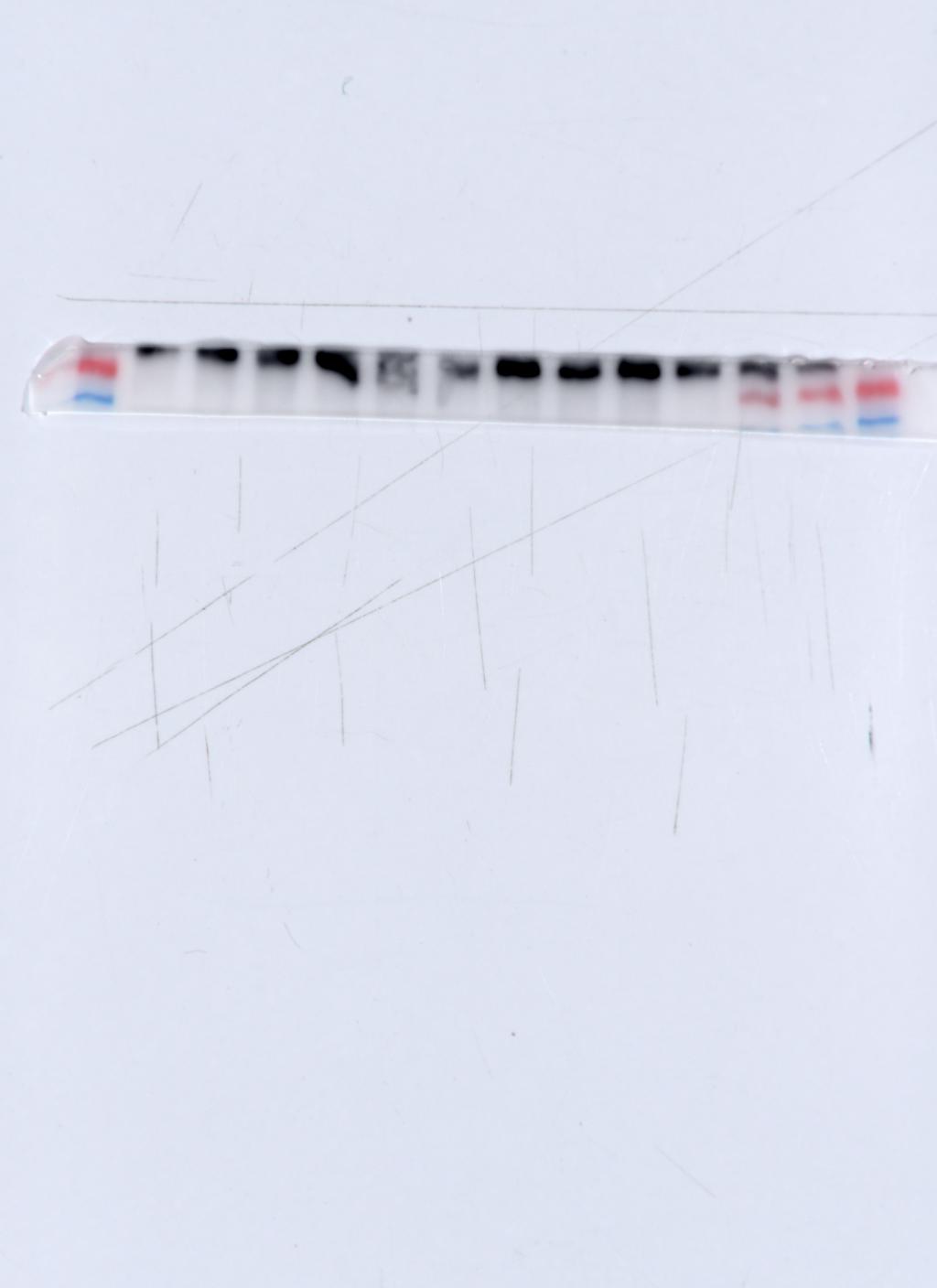

Supplement: Supplementary file 1 [file biomolecules-14-00592-s001.zip › Animal bands/HL VEGF/VEGF.jpg]

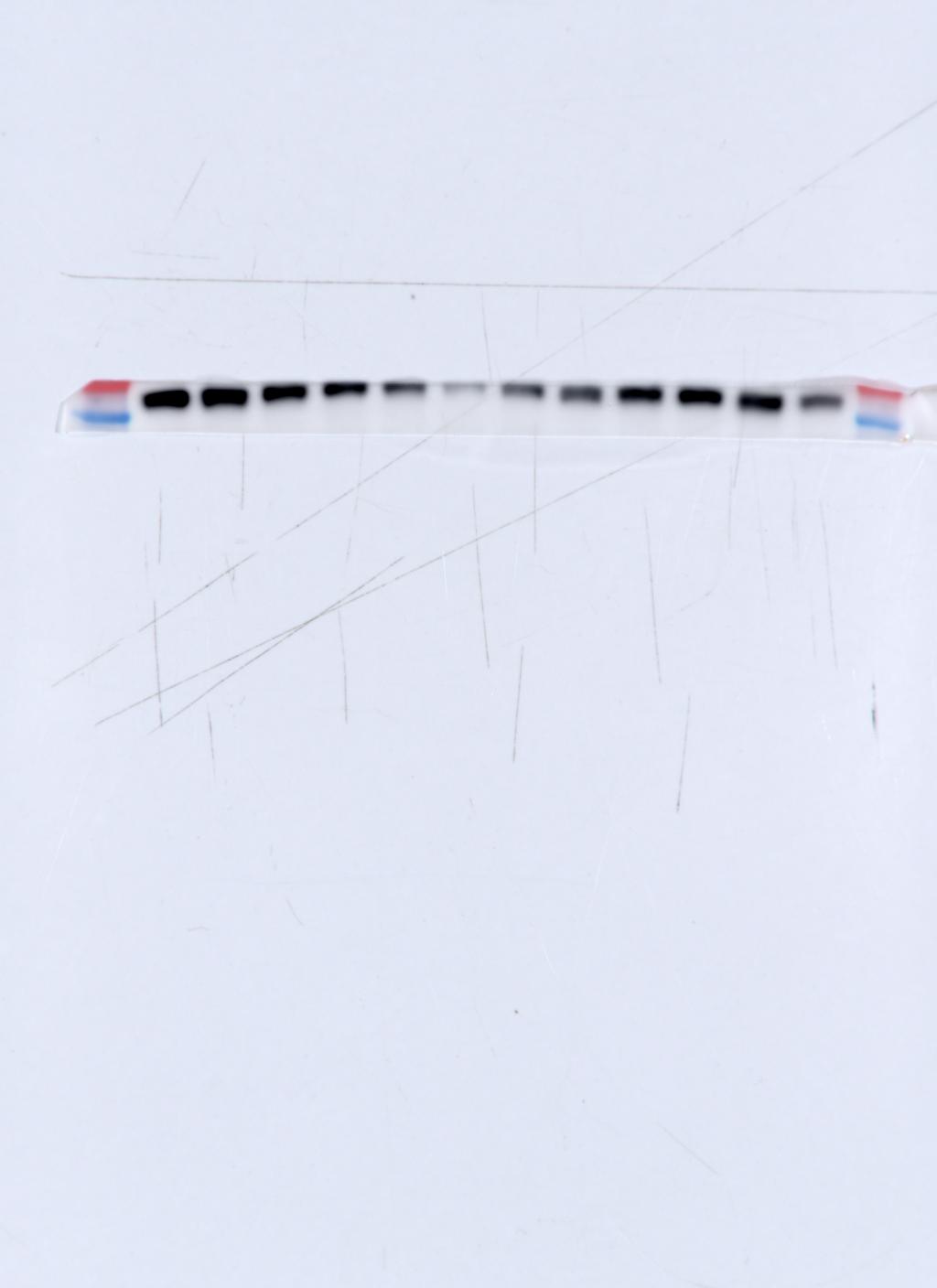

Supplement: Supplementary file 1 [file biomolecules-14-00592-s001.zip › Animal bands/HL akt/akt.jpg]

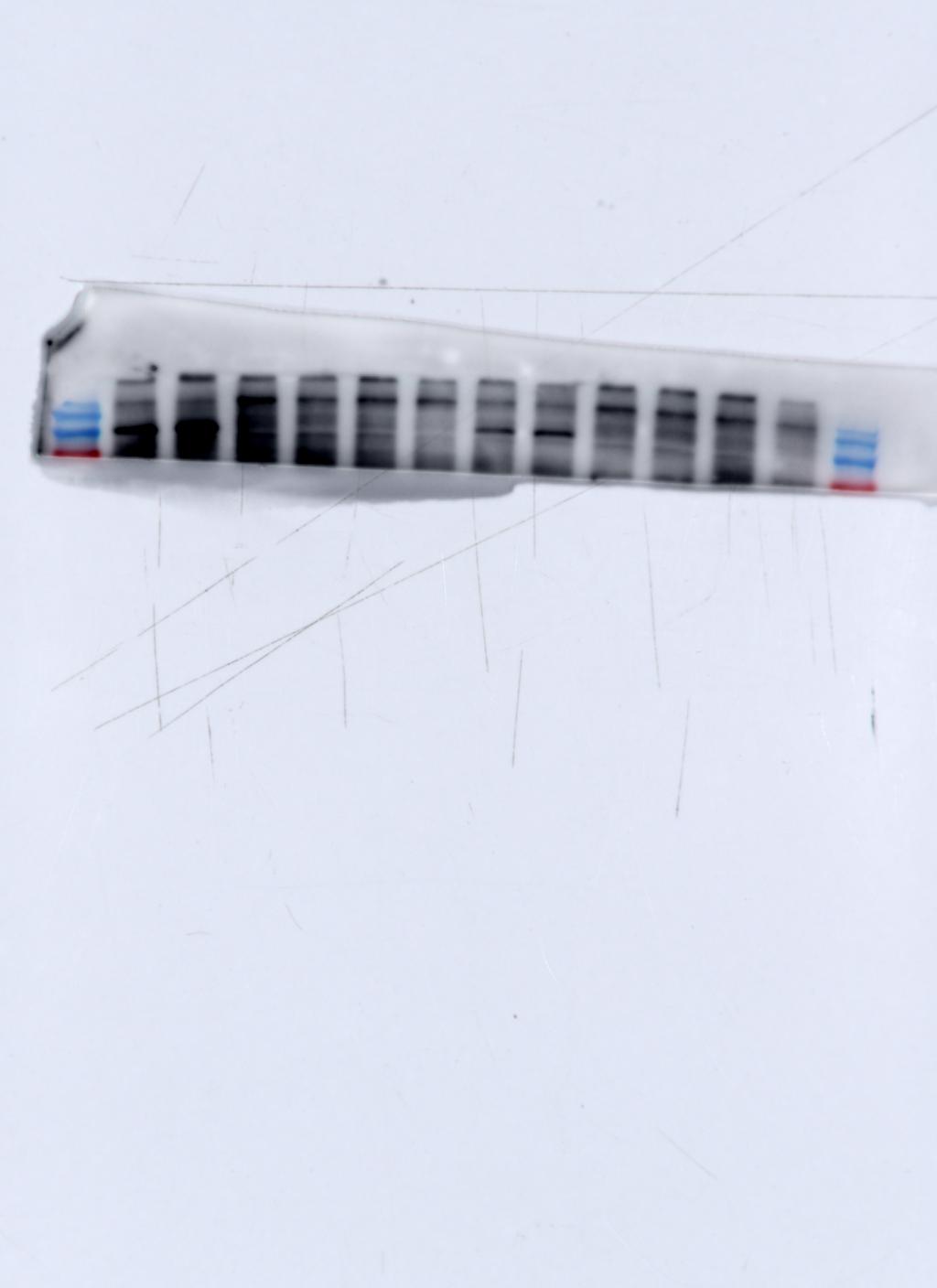

Supplement: Supplementary file 1 [file biomolecules-14-00592-s001.zip › Animal bands/HL p-enos/P-ENOS.jpg]

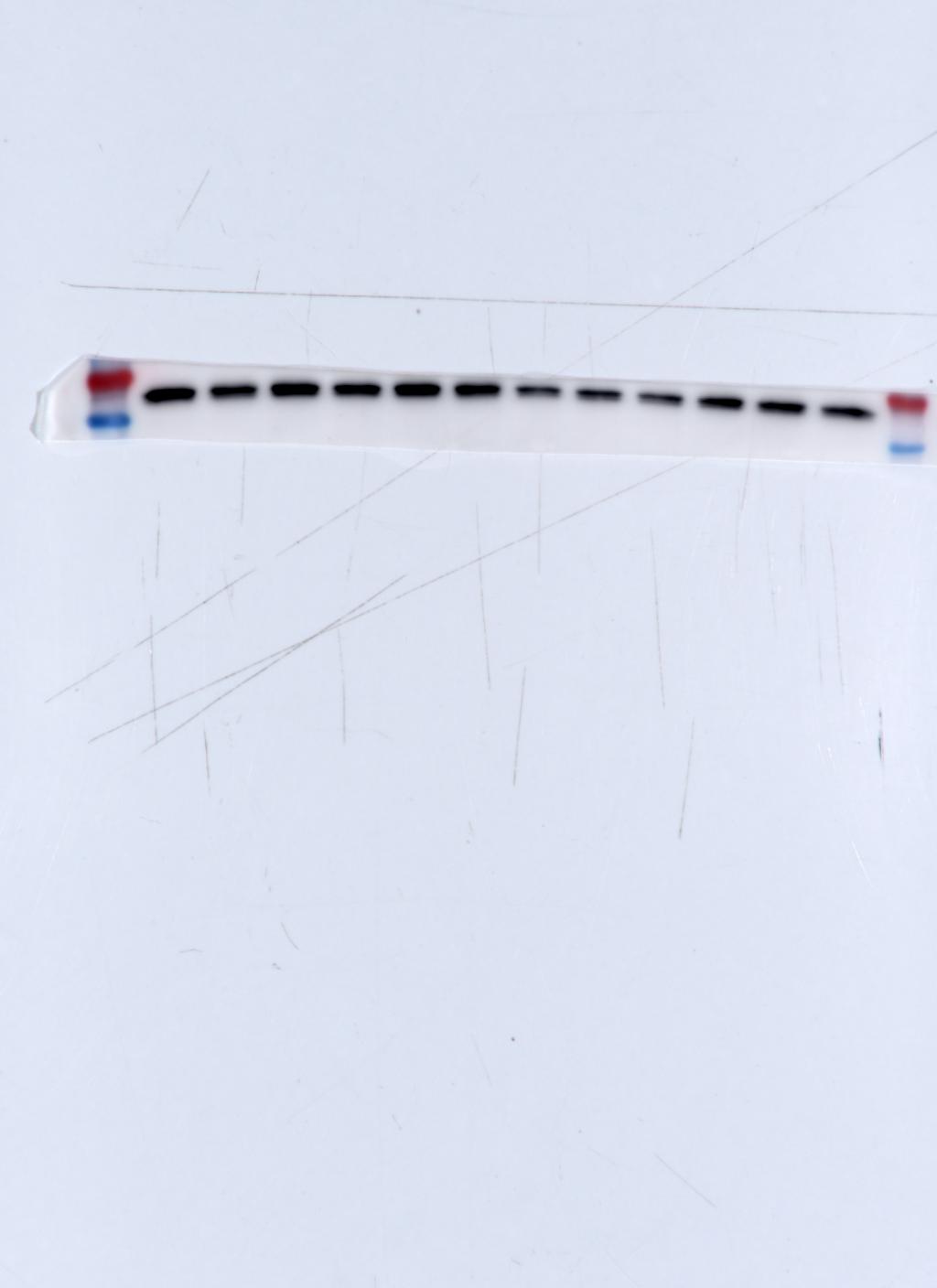

Supplement: Supplementary file 1 [file biomolecules-14-00592-s001.zip › Cell sieve concentration bands/AKT/akt.jpg]

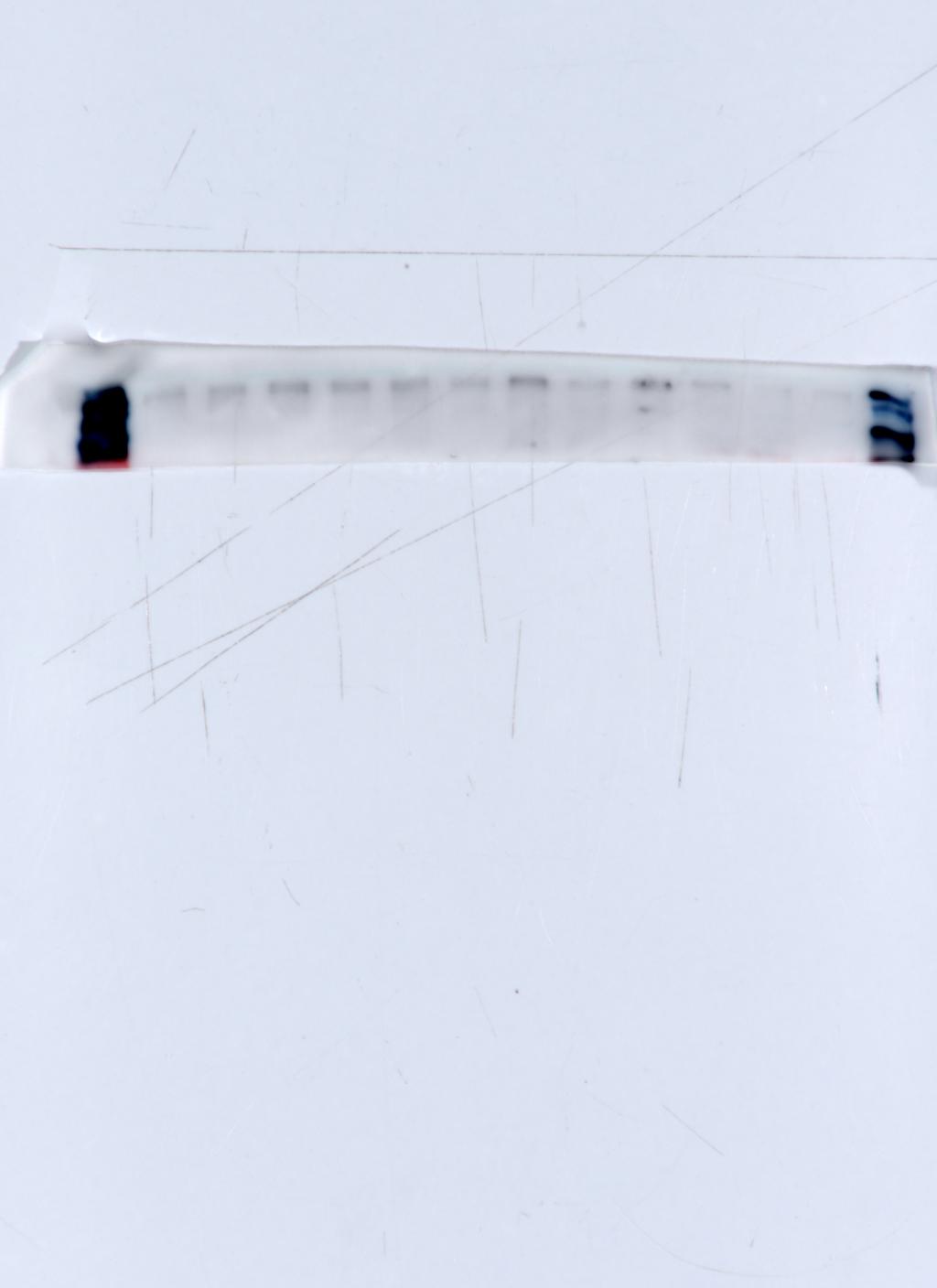

Supplement: Supplementary file 1 [file biomolecules-14-00592-s001.zip › Cell sieve concentration bands/ENOS/enos.jpg]

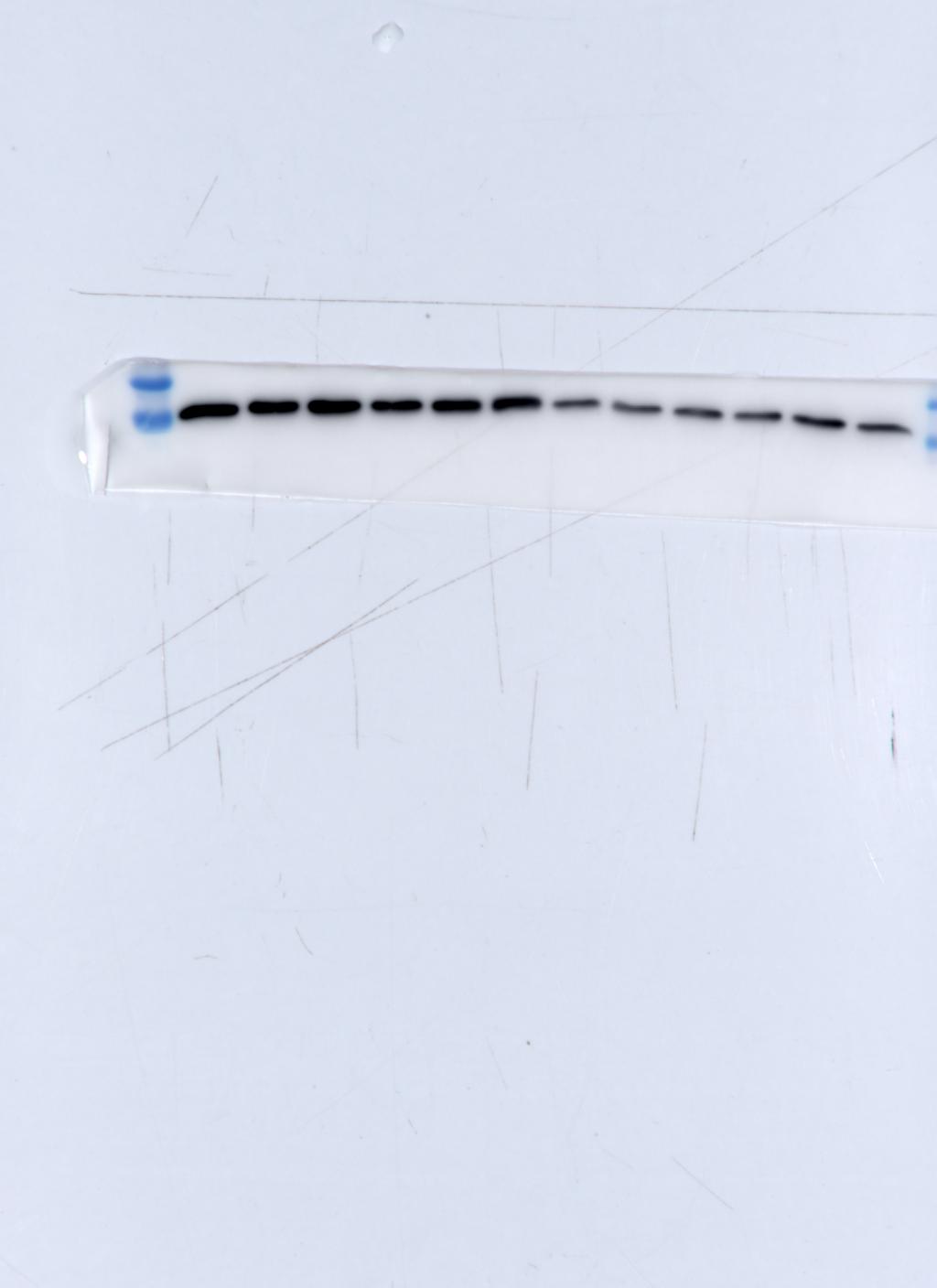

Supplement: Supplementary file 1 [file biomolecules-14-00592-s001.zip › Cell sieve concentration bands/GAPDH/GAPDH.jpg]

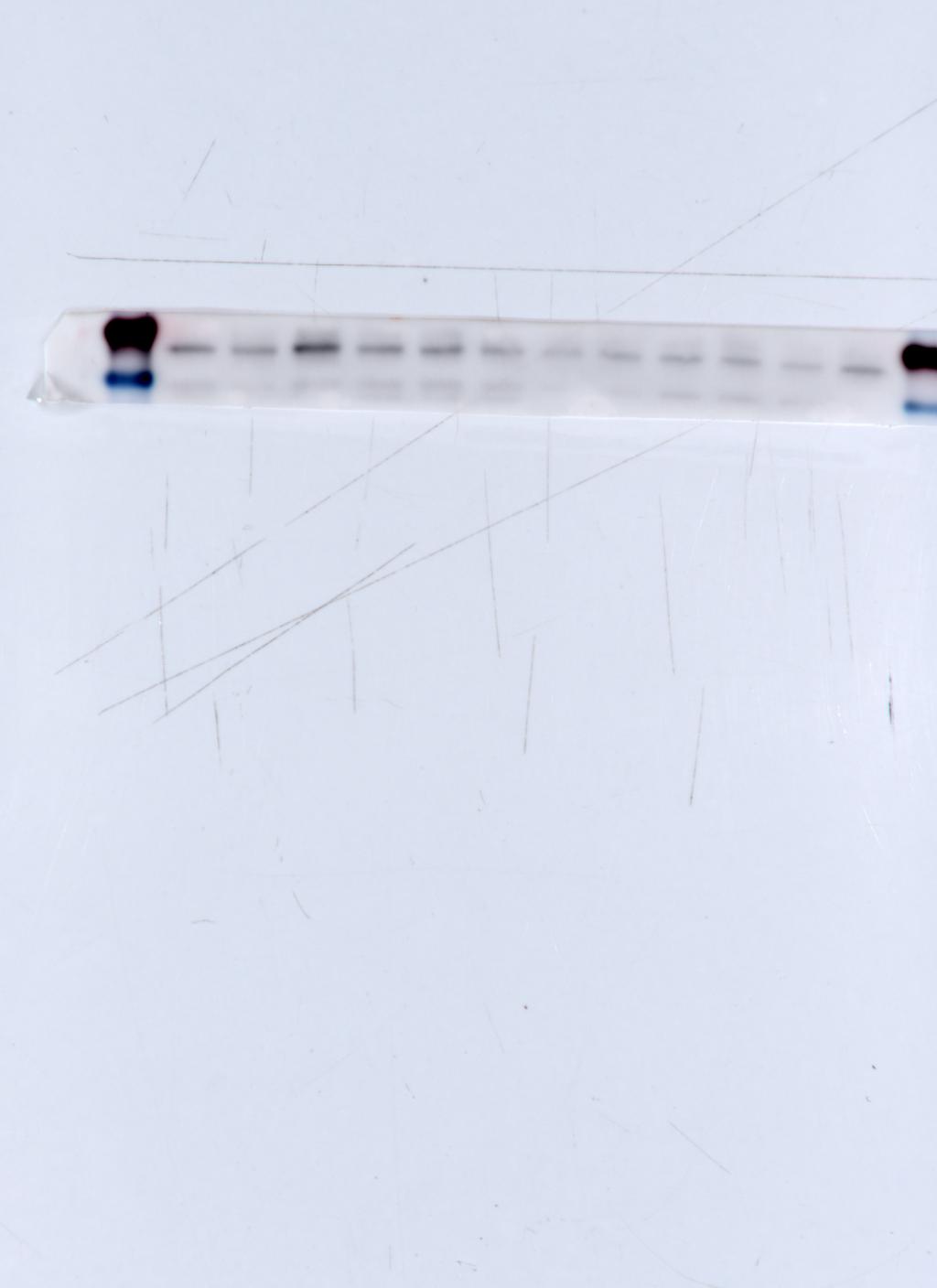

Supplement: Supplementary file 1 [file biomolecules-14-00592-s001.zip › Cell sieve concentration bands/P-AKT/p-akt.jpg]

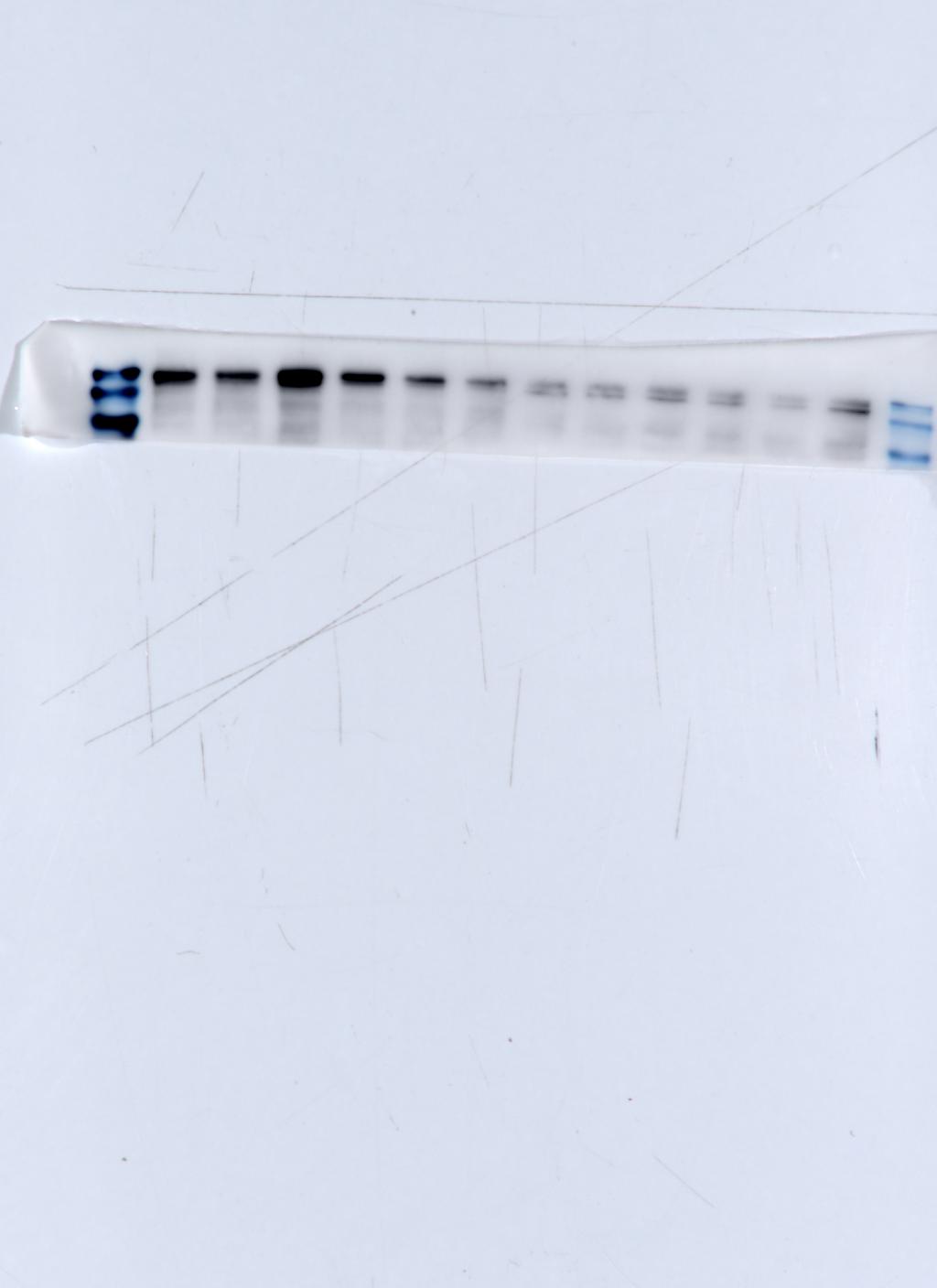

Supplement: Supplementary file 1 [file biomolecules-14-00592-s001.zip › Cell sieve concentration bands/P-ENOS/P-ENOS.jpg]

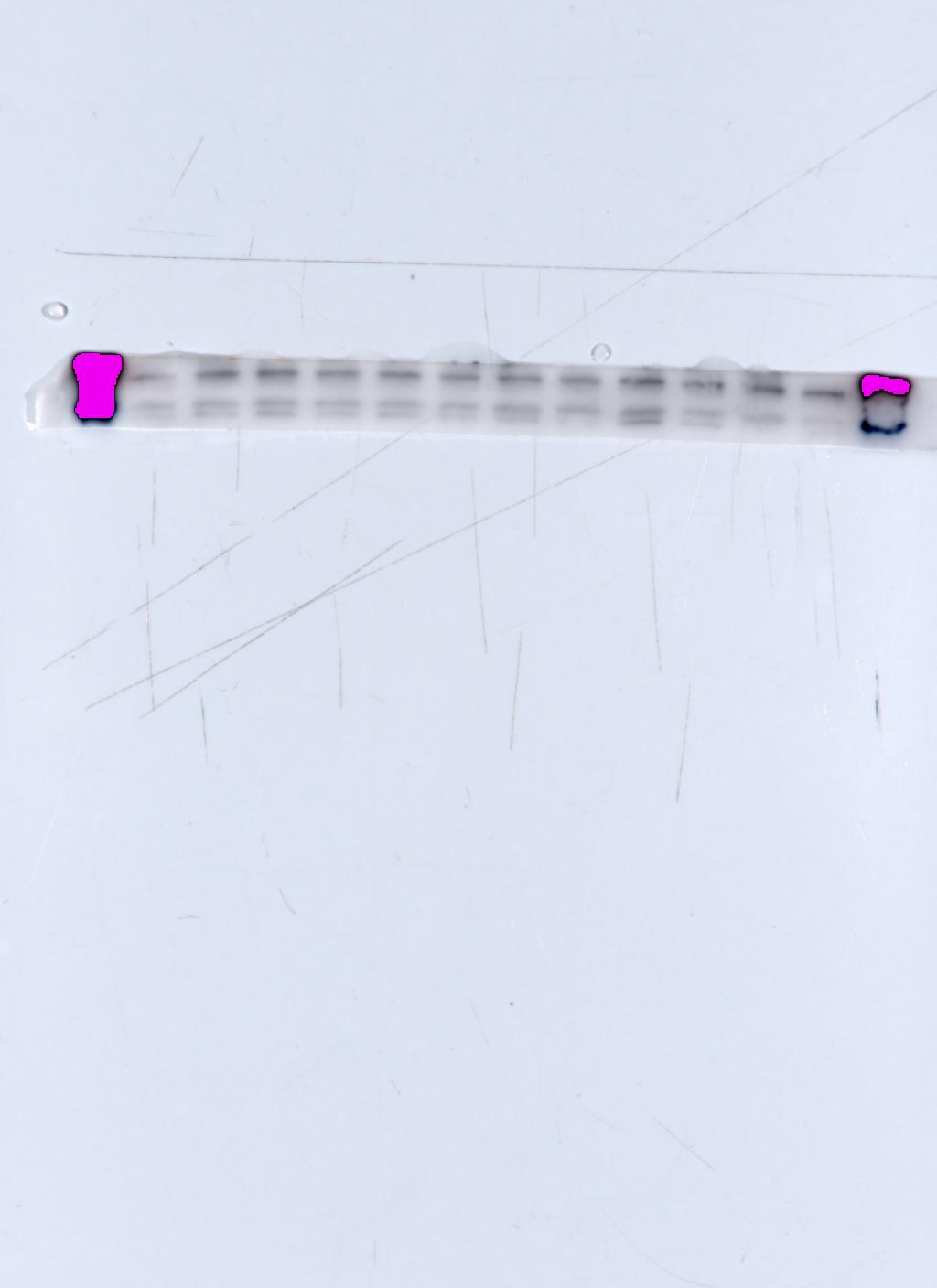

Supplement: Supplementary file 1 [file biomolecules-14-00592-s001.zip › Cell sieve concentration bands/vegf/vegf.jpg]

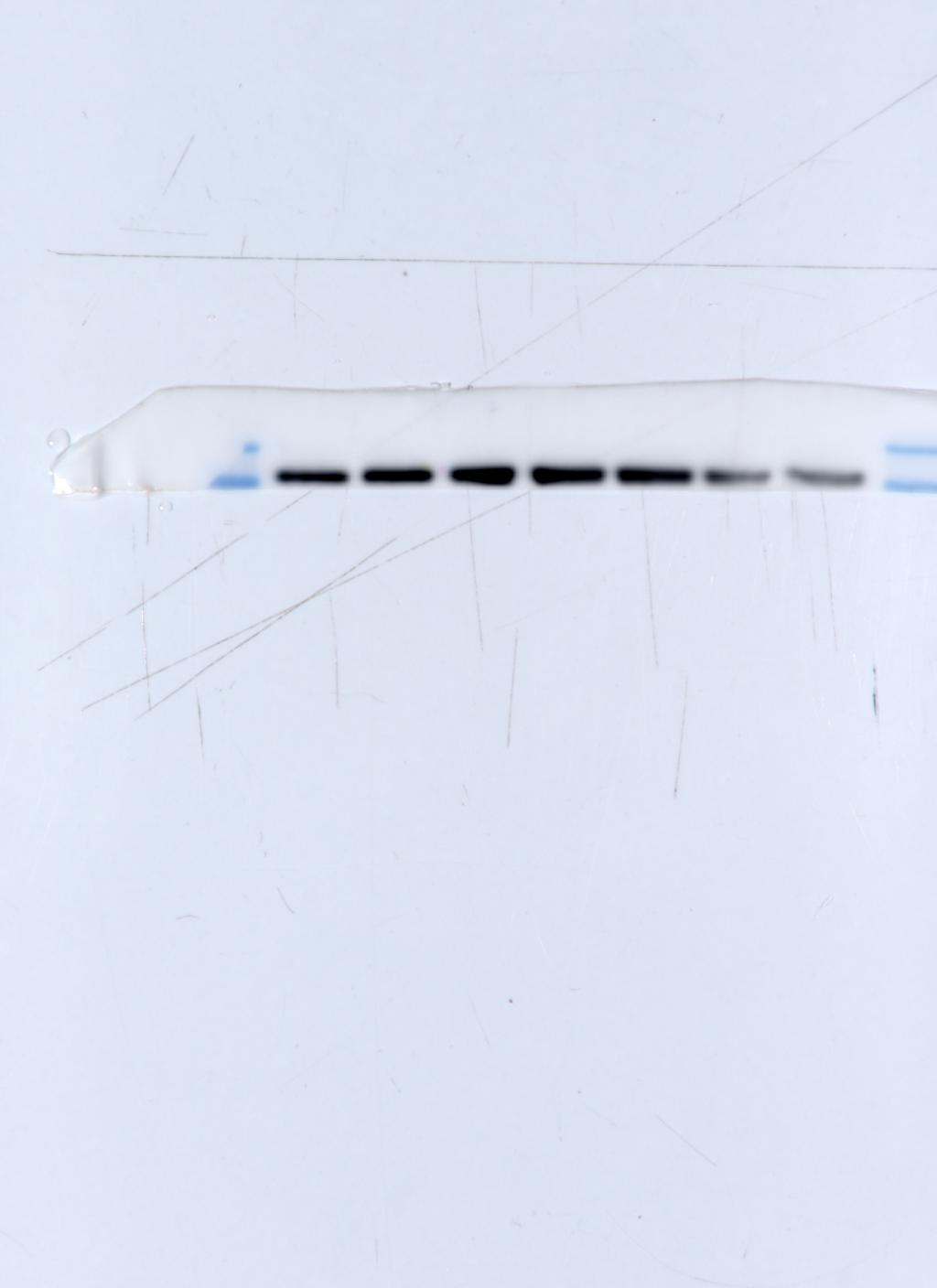

Supplement: Supplementary file 1 [file biomolecules-14-00592-s001.zip › Cellular mechanism bands/ENO--to the right/enos.jpg]

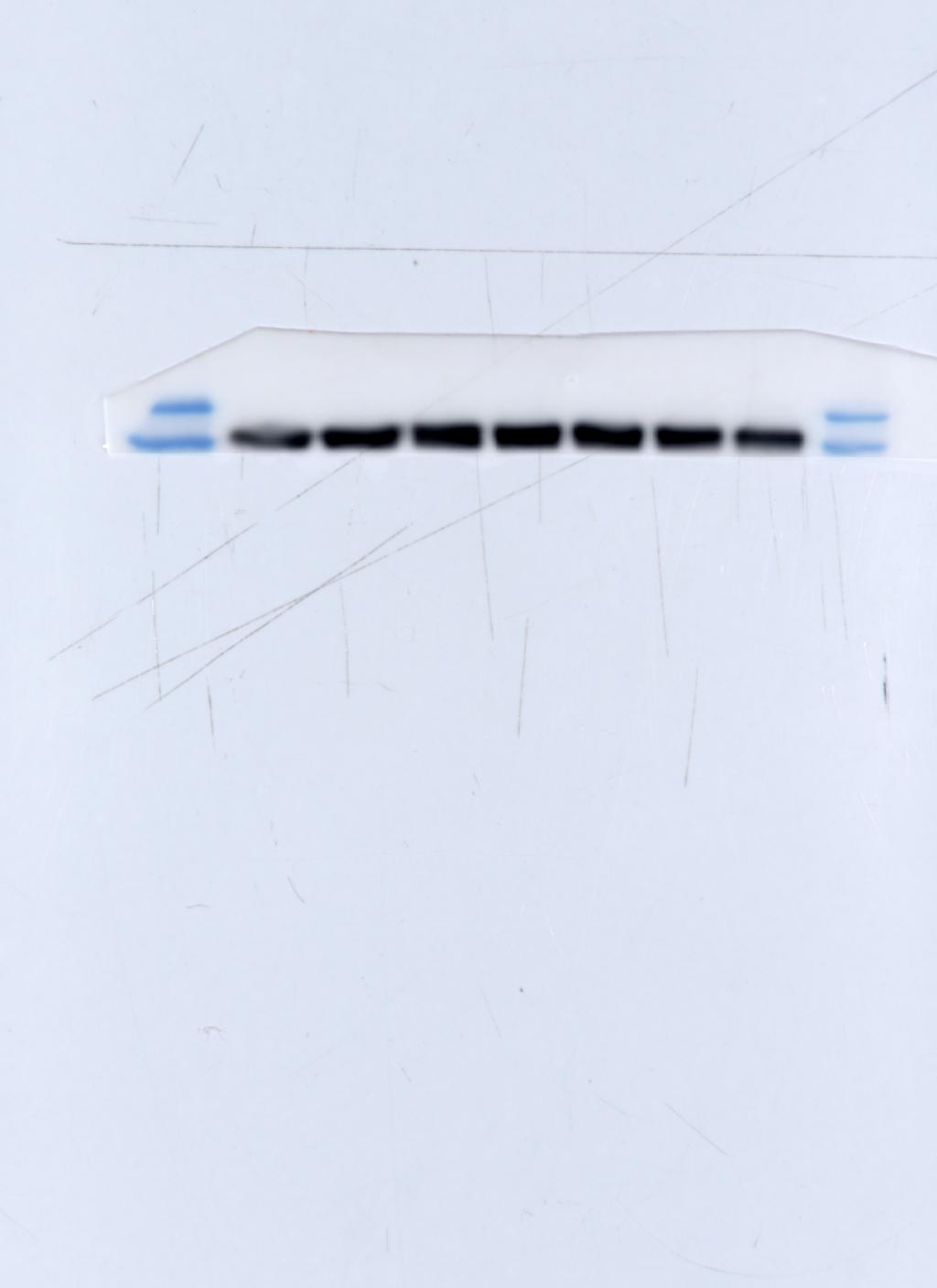

Supplement: Supplementary file 1 [file biomolecules-14-00592-s001.zip › Cellular mechanism bands/GAPDH2 --to the right/gapdh.jpg]

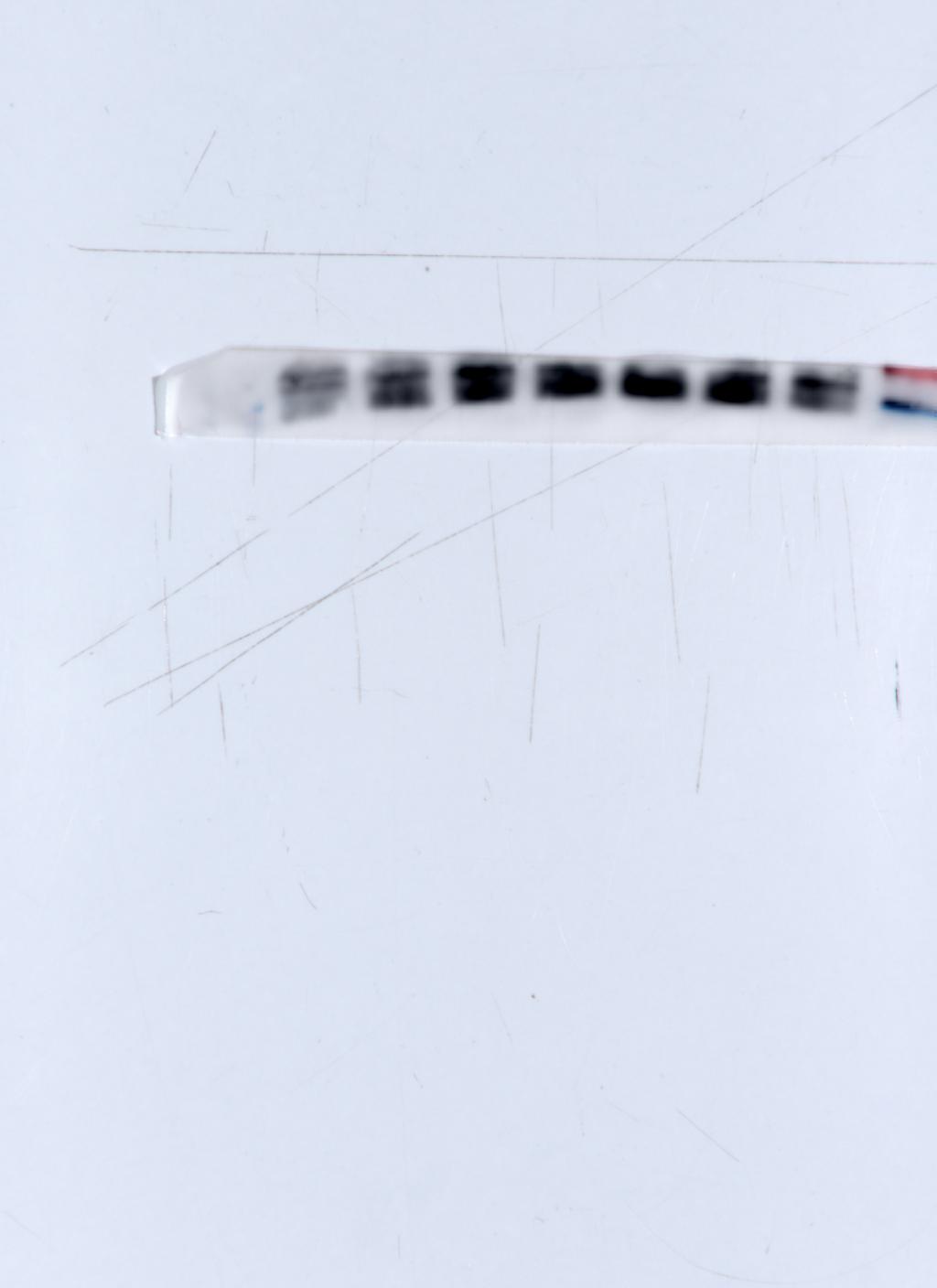

Supplement: Supplementary file 1 [file biomolecules-14-00592-s001.zip › Cellular mechanism bands/HL AKT--to the right/AKT.jpg]

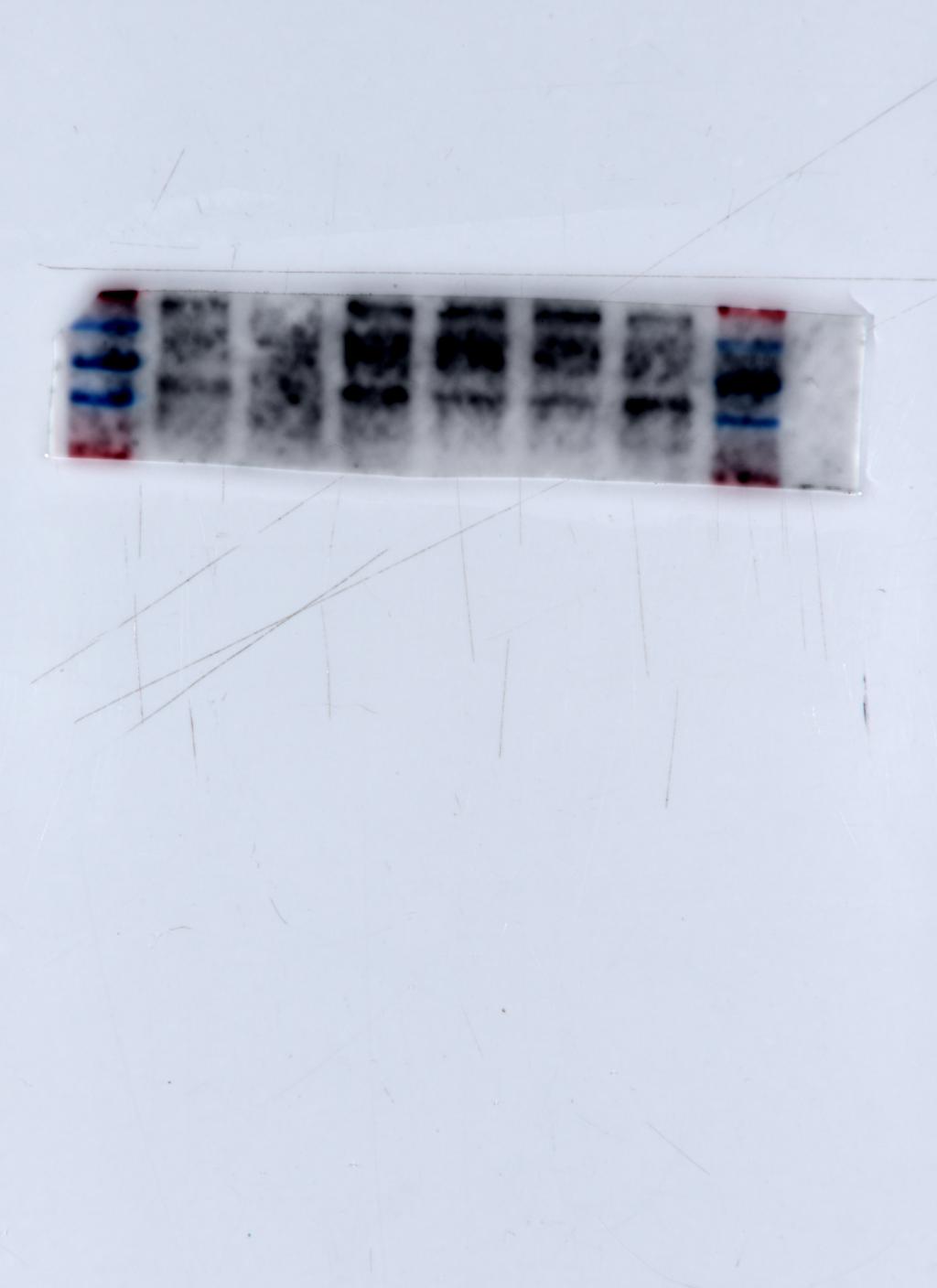

Supplement: Supplementary file 1 [file biomolecules-14-00592-s001.zip › Cellular mechanism bands/P-akt/p-akt.jpg]

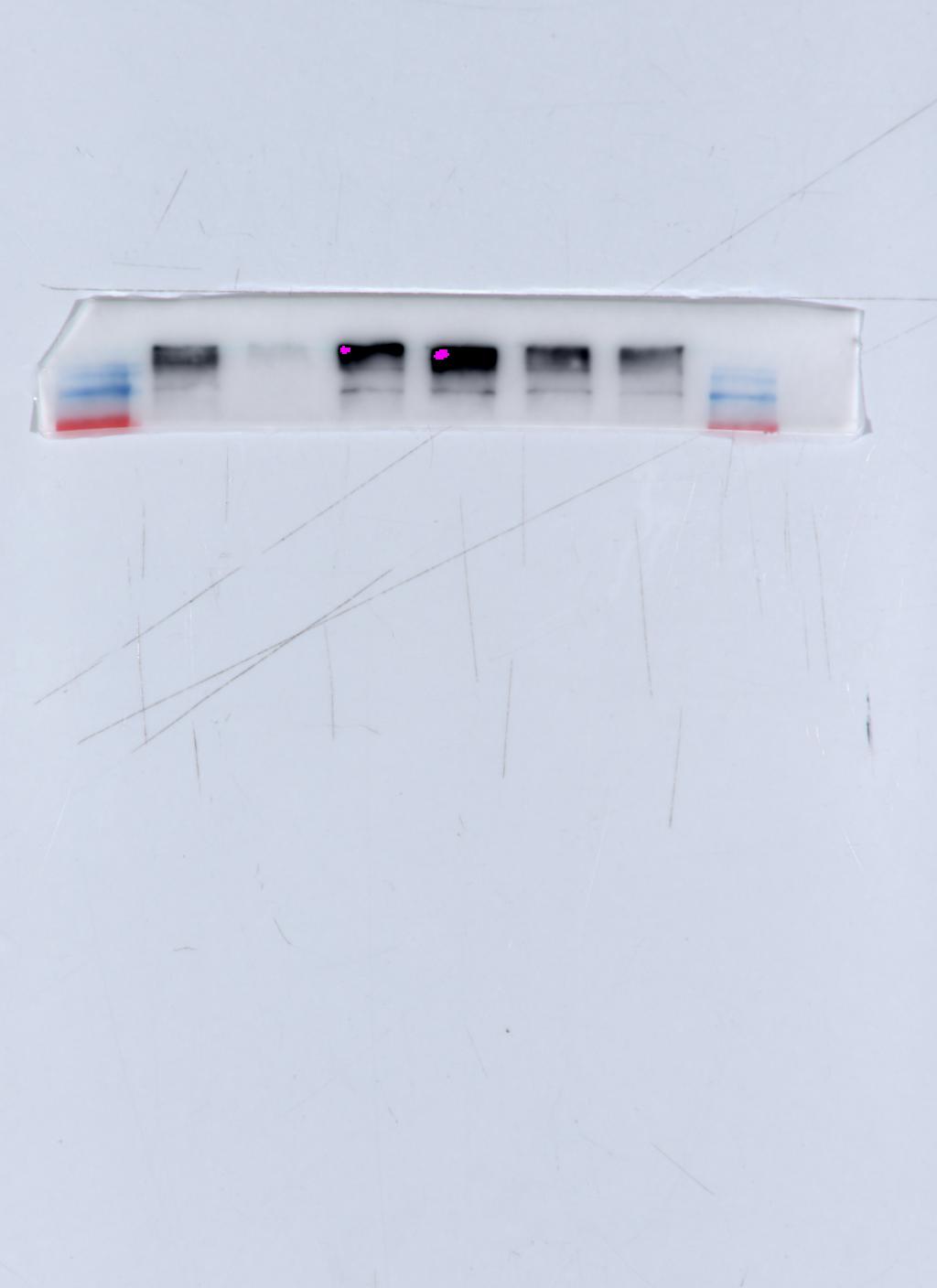

Supplement: Supplementary file 1 [file biomolecules-14-00592-s001.zip › Cellular mechanism bands/P-enos/P-enos.jpg]

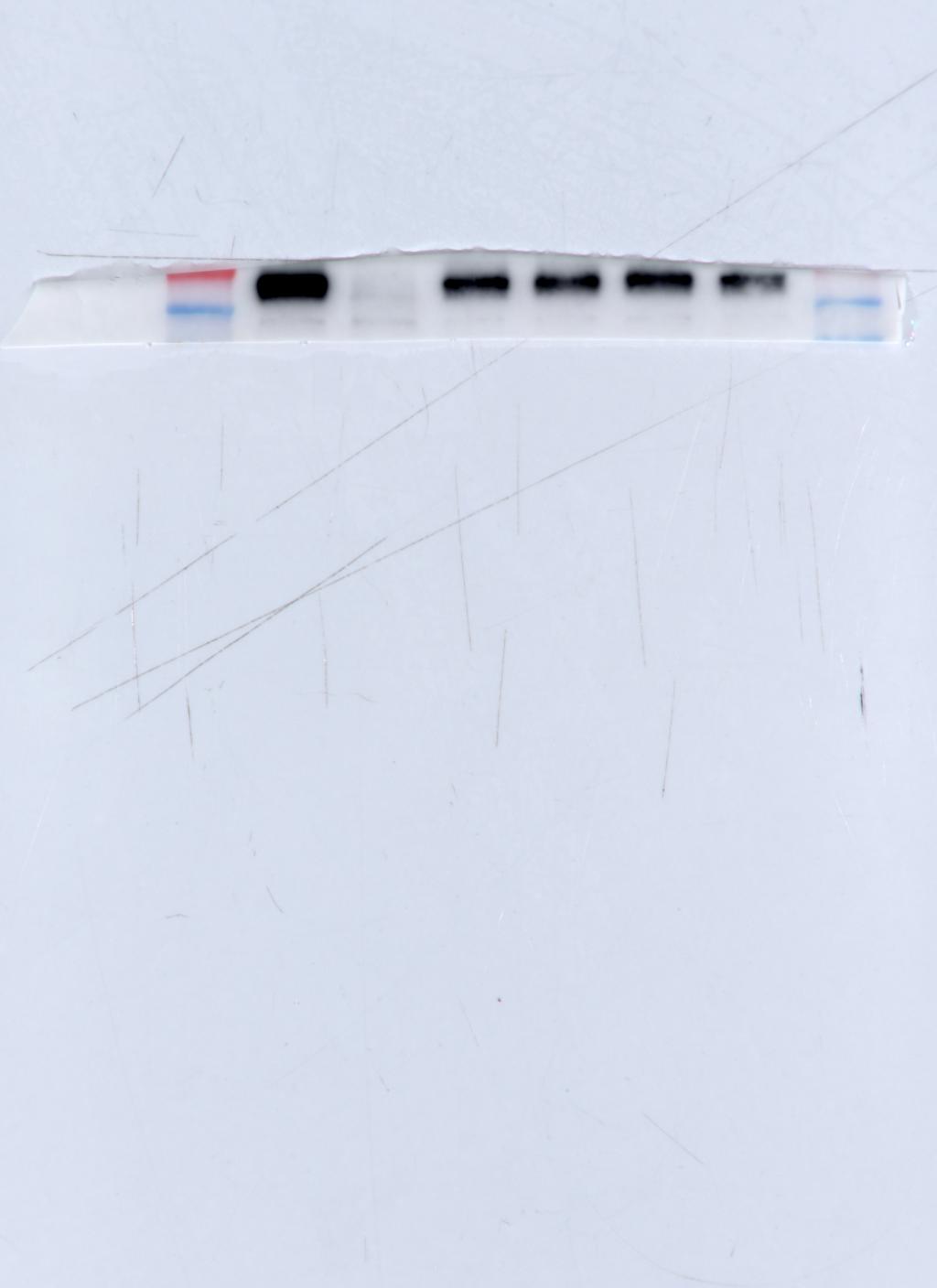

Supplement: Supplementary file 1 [file biomolecules-14-00592-s001.zip › Cellular mechanism bands/vegf/VEGF.jpg]
